# Diagnosis of Congenital Disorders of Glycosylation Type II Subtypes Through Comprehensive N-Glycan Profiling by Mass Spectrometry

**DOI:** 10.3390/ijms27146309

**Published:** 2026-07-15

**Authors:** Alan R. Mól, Nilza do C. Fontes, Savana C. L. Santos, Cynthia Costa e Silva, Gerson da S. Carvalho, Bruno J. C. B. Lima, Walquíria D. de Mello, Daniel R. de Carvalho, Eder A. Barbosa, Dirk J. Lefeber, Juliana F. Mazzeu, Jaime M. Brum, Guilherme D. Brand

**Affiliations:** 1 Laboratório de Síntese e Análise de Biomoléculas—LSAB, Instituto de Química, Universidade de Brasília, Brasília 70910-900, Brazil; alanmol@unb.br (A.R.M.); bioederr@gmail.com (E.A.B.); jaimebrum882@gmail.com (J.M.B.); 2 Laboratório de Genética Bioquímica, Rede Sarah de Hospitais de Reabilitação, Brasília 70335-901, Brazil; 3 Laboratório de Biologia Molecular, Rede Sarah de Hospitais de Reabilitação, Brasília 70335-901, Brazil; 4 Hospital de Apoio de Brasília, Secretaria de Saúde do Distrito Federal, Brasília 70684-831, Brazil; gdsilvacarvalho@gmail.com; 5 Hospital Universitário Júlio Muller, Faculdade de Medicina, Universidade Federal de Mato Grosso, Cuiabá 78048-902, Brazil; 6 Clínica de Pediatria do Hospital Infantil João Paulo II, Fundação Hospitalar do Estado de Minas Gerais, Belo Horizonte 30130-110, Brazil; 7 Translational Metabolic Laboratory, Department of Human Genetics, Donders Center for Brain, Cognition, and Behavior, Radboud University Medical Center, 6525 Nijmegen, The Netherlands; 8 Laboratório de Genética Clínica, Faculdade de Medicina, Universidade de Brasília, Brasília 70910-900, Brazil

**Keywords:** congenital disorders of glycosylation, inherited metabolic diseases, mass spectrometry, stable isotopic labeling, glycomics

## Abstract

Congenital disorders of glycosylation (CDG) are a group of inherited metabolic diseases rapidly growing due to the discovery of new subtypes. As with many genetic conditions, their diagnosis can be challenging, impairing proper patient care and causing additional suffering to patients and their families. We have developed an N-glycomics strategy that can provide insightful information towards diagnosing CDG type II (CDG-II). N-glycans released from the plasma of healthy individuals were labeled with deuterated iodomethane, mixed with samples from known or suspected CDG-II individuals, which were derivatized with standard iodomethane, and analyzed by liquid chromatography–mass spectrometry. After identification, relative quantification of 65 glycans was performed, revealing considerable alterations in the N-glycome of several patients. Notably, reduced fucosylation was observed in patients with FUT8-CDG and SLC35C1-CDG. Additionally, individuals with mutations in the *MAN1B1* gene exhibited increased amounts of hybrid and oligomannosidic structures, whereas patients with the Golgi homeostasis disorders *COG1*-CDG and *ATP6V0A2*-CDG presented marked increases in hypogalactosylated and hyposialylated structures. Multivariate statistical analysis indicated two undiagnosed patients with alterations similar to ATP6V0A2-CDG patients and another two with a profile similar to MAN1B1-CDG patients. Genetic sequencing (targeted gene panel or whole exome sequencing) of these undiagnosed patients revealed variants in the corresponding genes, confirming the diagnosis obtained from the N-glycomics analysis. Our results demonstrate how the analysis of total plasma N-glycans can be used to identify metabolic disorders and diagnose conditions based on their molecular effects on the glycoproteome.

## 1. Introduction

Congenital disorders of glycosylation (CDG) are a heterogeneous group of diseases first recognized in 1980 [1]. More than 200 disorders have been described to date, with approximately 57% of them involving N-glycosylation pathways [2]. The common biochemical basis is the defective incorporation or processing of glycans (complex sugars) into nascent proteins and lipids. The primary screening methodologies for CDG diagnosis are isoelectric focusing of transferrin (Tf-IEF), capillary electrophoresis (CE), and high-performance liquid chromatography (HPLC). The former is based on the isoelectric point of this protein, and the number of sialic acid residues in its glycan chains determines the isoelectric point [3]. An abnormal sialylation pattern found by this method points to one of the many known N-linked CDG subtypes, but does not provide enough details as to which one. False-positive results of Tf-IEF can occur due to transferrin polymorphisms [4] or due to primary or secondary liver dysfunction [5,6].

CDG subtypes involving N-glycan alterations arise from defects in the synthesis of the dolichol-linked glycan and its transfer to the protein in the endoplasmic reticulum (ER) or as it is processed into hybrid or complex glycans in the Golgi apparatus (GA). These subtypes are categorized as CDG type I and II, respectively, based on their Tf-IEF profiles [7]. Another way to group CDG subtypes is based on the type of affected glycans, in which an N-linked category has been proposed in the context of a broader CDG definition that also includes glycans not bound to proteins [2].

CDG-II results in complex alterations in serum protein-linked N-glycans, which can sometimes be difficult to distinguish from those of unaffected patients in Tf-IEF. A definitive diagnosis can be made directly by molecular methods such as single-gene sequencing, targeted gene panels, whole exome sequencing (WES), or whole genome sequencing (WGS). Nevertheless, despite the success of molecular approaches, biochemical data is still relevant, as each approach has its drawbacks [8]. Gene panels are restricted to target genes reported in the literature, and are less effective when investigating novel putative CDG subtypes. Additionally, as frequently observed in WES and WGS, a variant of unknown significance might be detected, which represents an inconclusive result that benefits from biochemical validations. A similar rationale applies to deep intronic variants, such as those described in TMEM165-CDG and ALG12-CDG cases, that need to be validated and that cannot be readily detected by WES [9,10]. Also, the increasing number of implicated genes complicates the creation of encompassing dedicated gene panels. Therefore, complementary techniques (e.g., the analysis of specific molecular biomarkers, intact glycosylated proteins, glycopeptides, or the released N-glycan structures by mass spectrometry) are often necessary for conclusive diagnosis [3,11]. A recent study has pointed to the complementarity of a joint glycomics and smMIPs sequencing approach in solving CDG-I cases [12].

Mass spectrometry (MS) is one of the most popular methodologies for the analysis of N-glycans, owing to its capacity to provide both qualitative and quantitative data for multiple analytes [13]. Different MS-based strategies can be used to diagnose CDG: (i) measuring the mass/charge ratio of a glycosylated reference protein, such as transferrin, and identifying its glycosylation profile [14,15]; (ii) analyzing free N-glycans released from plasma proteins or from a specific subset of proteins purified from whole blood [16,17]; and (iii) sequencing peptides hydrolyzed from plasma proteins along with their associated N-glycans (glycopeptides) [18,19]. MS has been used to describe characteristic N-glycan alterations for various CDG subtypes: accumulation of N4H3 and N4H3F1 in B4GALT1-CDG, hybrid structures in MAN1B1-CDG, and an overall decrease in fucosylated glycans in SLC35C1-CDG, as reviewed in the literature [14,20,21,22]. Regardless of the mode of analysis, extracting quantitative information is important, since the majority of CDGs result in alterations, sometimes subtle, in the relative concentrations of N-glycans already found in human plasma. Therefore, special care with multiple practical factors, such as mobile phase quality, column age, and instrument parameters, is needed to guarantee the reproducibility and accuracy of the method. For example, it has been demonstrated that equimolar mixtures of glycans can produce significantly different peak areas due to differences in the ionization efficiency of glycans based on their composition [23]. Analysis of permethylated glycans released from plasma proteins by mass spectrometry has been recommended as a technical standard for CDG diagnosis [24] due to the neutralization of charges of sialic acid, and fortunately, this technique can be combined with stable isotopic labeling (SIL) of a reference sample or a sample pool [25].

Although MS provides a great deal of information on glycosylated proteins or isolated glycans in the biological fluids of patients, data interpretation is often challenging. The most common approach is to obtain glycan profiles by MALDI-TOF MS or LC-MS and calculate relative peak areas to identify under- or overexpressed glycans [21]. The resulting glycan profile is interpreted considering the N-glycosylation pathway to identify potential enzymatic defects. Nevertheless, this is often a complicated task, as some CDG subtypes result in complex glycan profiles [22], which makes data interpretation difficult and restricted to skilled specialists.

In the present work, we apply multivariate statistical methods to the relative quantities of plasma N-glycans in three groups, namely healthy controls, diagnosed and suspected CDG-II patients, and their correlation with their molecular diagnosis. The goal is to develop a platform in which N-glycan profiles are more easily interpreted and can be used to guide the sequencing of putatively affected genes in CDG-II, narrowing the number of potentially affected genes and accelerating the diagnosis. This is especially relevant for CDGs in Brazil. CDGs are underdiagnosed worldwide due to the overlapping phenotype, and this situation is even more dramatic in Brazil due to its continental size and socioeconomic characteristics that limit access to specialized genetic centers. Hopefully, this platform can be used as an intermediary step between Tf IEF screening and final molecular diagnosis, providing further biochemical evidence of CDG. Additionally, this platform could aid in the description of novel CDG-II subtypes, the monitoring of therapeutic interventions in the plasma N-glycome of patients, and the functional validation of genetic variants in CDG subtypes that escape transferrin glycosylation analysis.

## 2. Results

### 2.1. Identification and Quantification of N-Glycans

**Figure 1:**
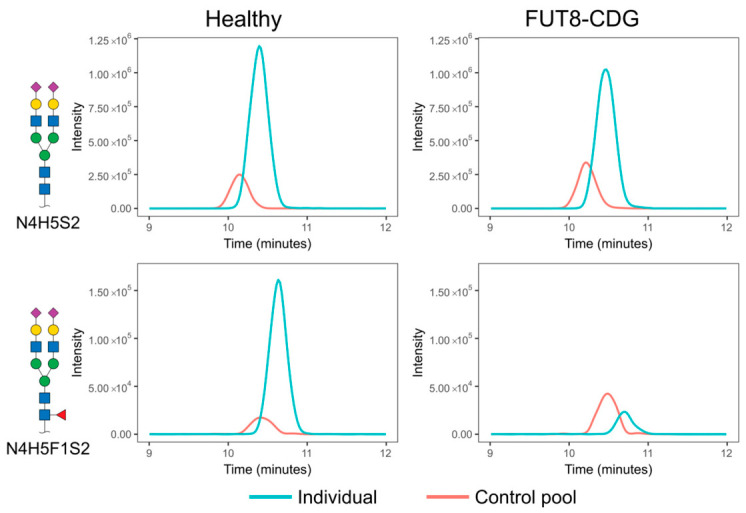
Extracted ion chromatograms (XICs) of two N-glycans, N4H5S2 (A2G2S2), and the N4H5F1S2 (FA2G2S2) obtained from the plasma of a healthy individual and a FUT8-CDG patient. The blue line corresponds to the intensity of the ion obtained either from the patient or the control, while the red line corresponds to the same data for the control pool. N4H5F1S2 was obtained by extracting an *m*/*z* of 1004.1501 from samples, while an *m*/*z* of 1045.4074 (the deuterated N4H5F1S2) was extracted from the control pool. N4H5S2 was obtained by extracting an *m*/*z* of 1407.6859 from samples, while an *m*/*z* of 1466.5531 (deuterated N4H5S2) was extracted from the control pool. N: *N*-Acetyl glucosamine, H: hexose, F: fucose, S: sialic acid.

N-glycans were obtained from the plasma of controls, CDG-II patients, and putatively affected CDG-II individuals, and submitted to LC-MS/MS analysis in positive mode in an information-dependent acquisition (IDA) experiment along with the control pool. A stable isotope labeling (SIL) strategy was used for the differential tagging of N-glycans, as described elsewhere [16]. Chromatography solvents were enriched with Na^+^ ions using NaOH as the solvent phase modifier to reduce the complexity of the sample by preventing the formation of H^+^, NH_4_^+^, and K^+^ adducts. As expected, multiply charged sodium adducts were predominantly detected in MS analyses, and ions matching known N-glycan *m*/*z* ratios were annotated. Sixty-five unique N-glycan compositions were consistently detected across all samples, of which 62 were annotated by the GRITS Toolbox and 31 by the in-house script. The list of all N-glycan compositions and their putative structures, as interpreted from their corresponding fragmentation spectra, is available in Table S1. Figure 1 presents the extracted ion chromatogram (XIC) of some selected glycan compositions for a FUT8-CDG patient and a healthy control, both compared to the mass-tagged control pool. It is straightforward to observe that the ion corresponding to N4H5F1S2 (FA2G2S2 in Oxford notation) presents a lower intensity in FUT8-CDG when compared to a control individual, which is not true in the case of the non-fucosylated bi-antennary counterpart N4H5S2 (A2G2S2). This indicates that while N4H5F1S2 in the FUT8-CDG patient is less abundant than in the healthy individual, the N-glycan N4H5S2 is unaltered. Similarly to the case presented above, XICs were obtained for all ions consistent with annotated glycan compositions for all LC-MS/MS acquisitions, generally considering the ion with the highest intensity among the detected charge states and isotopes. The retention time of glycans was correlated with their *m*/*z* ratio, as suggested in the literature [26]: higher *m*/*z* led to higher retention in a C18 column, as depicted in Figure S1. This correlation was used to identify inconsistencies in glycan annotation. Inspection of mass spectra revealed that for some compositions, there was an overlap between ions belonging to different glycans, which were not fully separated during chromatography. For example, the N-glycan N2H7 (M7) doubly charged ion ([M + 2Na]^2+^) presented an *m*/*z* of 1005.4837, which coincides with the fifth isotopologue of N4H5F1S2 (FA2G2S2)’s [M + 3Na]^3+^ ion (Figure S2a). Because N4H5F1S2 is more abundant in human plasma, quantitation using N2H7’s first isotopologue could lead to overestimation, considering they have similar retention times (Figure S2b). For such cases, the most intense isotopologue without a detectable overlap with other N-glycan ions was used for quantification, a procedure that was performed both for the permethylated and perdeuterated glycans.

The areas of the 65 N-glycans evaluated in our method across all individuals were extracted, divided by the respective reference N-glycans obtained from the control pool, log-transformed, and listed in table format (Table S1). Comparison of each N-glycan between patients and the control pool is available in Figures S4–S19. A principal component analysis (PCA) was applied to these data, which resulted in 13 PCs explaining approximately 85% of the data variance. The data demonstrates that the controls form a central volume in the first PCs, for which a 95% confidence interval ellipse can be drawn, allowing the determination of statistically relevant alterations from normality in this space. It is relevant to highlight that PCA is a non-supervised statistical method, and therefore, no prior classifications, such as control, suspected CDG-II patient, or reference CDG patient, are supplied to the model. The principal component locations of known CDG-II patients allowed the identification of some groups among CDG subtypes, as presented below.

### 2.2. Fucosylation Defects: FUT8-CDG and SLC35C1-CDG

**Figure 2:**
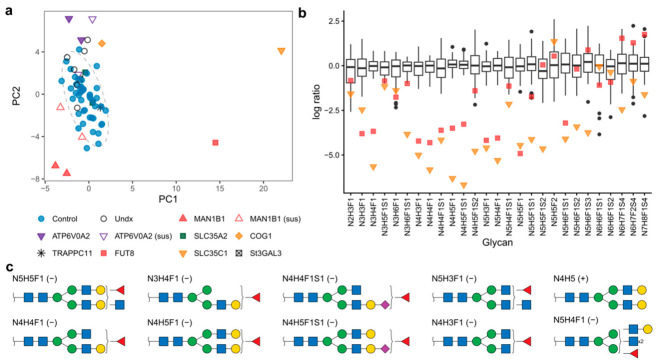
Fucosylation-related disorders lead to marked N-glycome alterations. (**a**) Projection of all analyzed cases in principal components 1 and 2. Component 1 is influenced by fucosylated glycans, separating FUT8- and SLC35C1-CDG from the remaining cases. The dotted ellipse delimits the 95% confidence region of the control cases. (**b**) Box-plot of the log-transformed N-glycan ratios, highlighting the extent of changes in FUT8- and SLC35C1-CDG in relation to healthy controls for some selected glycan compositions. (**c**) Structures of N-Glycans with the largest absolute loadings on PC1. The signal after the glycan composition indicates its loading signal on the component.

The first principal component (PC1, 19.9% explained variance) clearly separated two patients (FUT8-CDG and SLC35C1-CDG) from the remaining cases (Figure 2a). These conditions are known to considerably reduce the abundance of fucosylated N-glycans (Figure 2b), which explains both the magnitude of the effect on PC1 as well as the fact that both presented similar trends. Analysis of the loadings of PC1 sheds light on this matter: The 19 N-glycans with the largest negative loadings on this component are all fucosylated, and some selected compositions, along with their putative structures, are listed in Figure 2c. Six other fucosylated glycans had loadings with a negative value.

Fucosylated N-glycans were reduced in both FUT8- and SLC35C1-CDG patients when compared to healthy controls. However, the SLC35C1-CDG patient showed more pronounced alterations—both quantitatively, with a higher median deviation, and qualitatively, with a greater number of significantly altered glycans. This difference can be attributed to the metabolic pathways characteristic of each disease. The fucosyltransferase 8 (FUT8) enzyme is responsible for adding a fucose to the first N-acetyl glucosamine (GlcNAc) of the N-glycan core but does not affect fucosylation on the antennae. Therefore, it is expected that patients with FUT8-CDG will still have normal levels of N-glycans fucosylated in the antennae, despite the lower overall abundance of these in relation to core-fucosylated glycans. GDP-fucose transporter 1 (SLC35C1) is a membrane protein that transports guanosine diphosphate fucose (GDP-fucose) units from the cytoplasm into the Golgi apparatus. The absence of or reduced activity of this transporter affects the fucosylation of the glycan core and antennae alike due to the limited availability of GDP-fucose.

**Figure 3:**
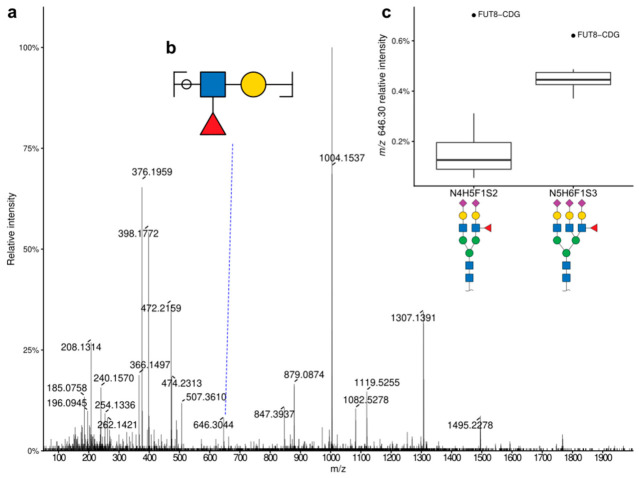
Identification of antennary fucosylation using a diagnostic fragment ion. (**a**) MS/MS spectrum of the N-Glycan N4H5F1S2 from a FUT8-CDG patient. (**b**) Putative structure of the fragment generated from the fragmentation of the N-glycan antennae, with *m*/*z* 646.3044. (**c**) Relative intensities of the diagnostic fragment in the patient with FUT8-CDG compared to controls for two different N-Glycans (N4H5F1S2 and N5H6F1S3). This patient has a significantly higher relative intensity of this diagnostic fragment, which is not observed in the patient with SLC35C1-CDG.

The fragmentation spectra of N4H5F1S2 from the FUT8-CDG and SLC35C1-CDG patients illustrate how MS/MS profiles can be used to differentiate these two subtypes by evaluating ions produced with specific fucosylation locations (Figure 3a). N4H5F1S2 is the most abundant fucosylated glycan in human plasma, and despite the overall low abundance of this glycan in these two CDG subtypes, its fragmentation for both patients resulted in reliable MS/MS spectra. The relative area of the fragment *m*/*z* 646.3045 was extracted from the MS/MS spectra of [M + 3Na]^3+^ = 1004.1501 Da from all LC-MS/MS analyses. This ion is indicative of a fucose in the antennae location, given that the composition GlcNAc(Fuc)-Gal is exclusively obtained from these structures (Figure 3b). It is observed that for controls and the other CDG subtypes evaluated herein, the intensity of this ion is generally low, which indicates that core-fucosylation is the most common glycoform for N4H5F1S2 in our analyses. Nevertheless, for the FUT8-CDG patient, the residual N4H5F1S2 quantified from plasma proteins corresponds mostly to the glycoform where fucose is located in the antennae, and not on the core, as expected for FUT8 deficiency (Figure 3c). The same trend is observed for another, less abundant fucosylated glycan, N5H6F1S3, precursor [M + 3Na]^3+^ = 1274.2833 Da (Figure 3c). Thus, we propose that this ion can be used to differentiate FUT8-CDG and SLC35C1-CDG patients in these fucosylated glycans.

In addition to the expected reduction in fucosylated glycans, increased levels of some non-fucosylated glycans were also observed in FUT8- and SLC35C1-CDG patients. These contributed significantly to PC1 and similarly to fucosylated structures (Figure 1). Examples of glycans that presented this behavior are N4H3 (A2G0) and N4H5 (A2G2). Analysis of the glycosylation pathways sheds light on these alterations: N4H3 corresponds to the composition of the substrate of the FUT8 enzyme. The relative increase in N4H5 can be interpreted as secondary to N4H3. Altogether, the data described herein demonstrate that the proposed platform for plasma N-glycan analyses, followed by multivariate statistics, can confidently separate patients with fucosylation disorders from normal controls and other CDG subtypes. However, no undiagnosed patient in our cohort presented a compatible N-glycan pattern, which is not surprising considering these subtypes do not usually present altered Tf IEF profiles, and broader inclusion criteria would be necessary for these individuals to be included. Finally, it can be stated that the variants found in these two patients, whose significance was marked as uncertain for the FUT8-CDG patient and likely pathogenic for the SLC35C1-CDG patient, are biochemically relevant and indeed pathogenic.

### 2.3. Accumulation of Hybrid N-Glycans and Identification of MAN1B1-CDG

**Figure 4:**
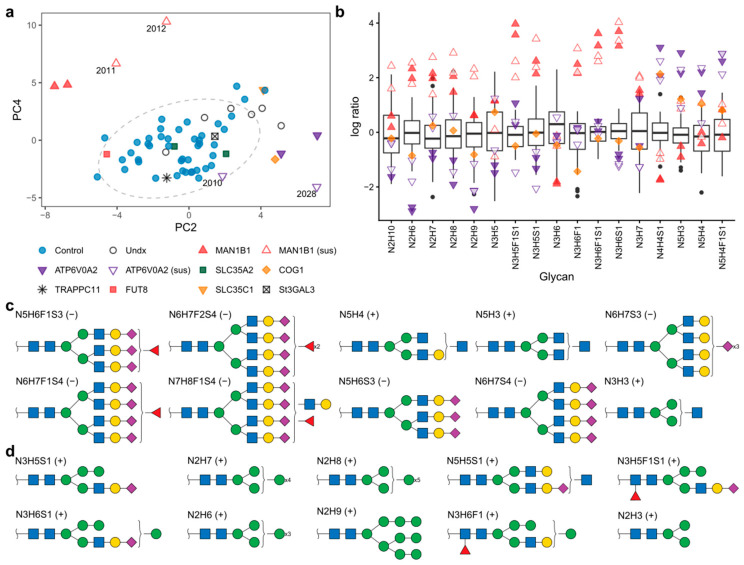
The score plot of principal components 2 and 4 can be used to identify CDG subtypes linked to the accumulation of hybrid N-glycans and immature structures. (**a**) Projection of all analyzed cases in principal components 2 and 4. The dotted ellipse represents the 95% confidence region of the control cases. MAN1B1-CDG cases have a negative projection in PC2 and a positive projection in PC4, and two suspected CDG-II patients co-localized with them. Golgi homeostasis defects, such as ATP6V0A2- and COG1-CDG, have a positive projection in PC2 and a negative projection in PC4. Also, two CDG-suspected patients co-localized in this area. (**b**) Box plot of the log-transformed N-glycan ratios, highlighting the extent of changes in ATP6V0A2- and COG1-CDG in relation to healthy controls for some selected glycan compositions. (**c**) Structures of N-Glycans with the largest absolute loadings on PC2. (**d**) Structures of N-Glycans with the largest absolute loadings on PC4. The signal after the glycan composition indicates its loading signal on the component.

The combination of principal components 2 (13.99% explained variance) and 4 (11.48% explained variance) separated a group of four patients from the cohort (Figure 4a), two of them being known MAN1B1-CDG cases and two being undiagnosed patients (2011 and 2012, siblings). In-depth analysis of glycans on these four samples reveals significant alterations in hybrid structures, which are overabundant in relation to controls (Figure 4b). The two suspected CDG-II patients presented high relative quantities of the hybrid N-glycans N3H5F1S1, N3H6S1, and N3H6F1S1 (Oxford, FM4A1G1S1, M5A1G1S1, and FM5A1G1S1), among others. This is a hallmark of MAN1B1-CDG, as described herein and in the literature [27]. Indeed, hybrid N-glycans presented high loadings in both PC2 and PC4 (Figure 4c). Accumulation of hybrid structures is consistent with MAN1B1-CDG, since the enzyme alpha-1,2-Mannosidase (MAN1B1) cleaves a mannose (Man) from one of the antennae during the N-glycans synthetic route, a step which is required prior to the addition of one of the GlcNAc present in the antennae of complex N-glycans [28]. Without this step, an increase in hybrid N-glycans is expected, although MAN1B1-CDG patients usually present normal levels of complex glycans, which can be a consequence of residual activity of the enzyme or the product of other synthetic pathways. The co-localization of these two suspected CDG-II patients with known cases of MAN1B1-CDG in our platform prompted us to confirm their diagnosis by gene sequencing.

The DNA samples of the two suspected MAN1B1-CDG patients were extracted and submitted to whole exome sequencing (WES) (Table 1). For both, a homozygous variant on the *MAN1B1* gene was found (c.1735dup p.Gln579Profs*75), confirming the diagnosis of MAN1B1-CDG. This variant was detected with high confidence and is marked as likely pathogenic in ClinVar and pathogenic in Franklin. To the authors’ knowledge, this is the first time this variant has been described; thus, it is novel. Notably, these patients are siblings of approximately 50 years old and did not have a diagnosis of their condition up until our findings, illustrating the shortage of methods for the diagnosis of CDG in Brazil.

### 2.4. Golgi Homeostasis Defects: ATP6V0A2-CDG and COG1-CDG

Principal components 2 and 4 also showcased four patients in the opposite quadrant of the MAN1B1-CDG patients, two of whom were known ATP6V0A2-CDG patients, one COG1-CDG, and one undiagnosed patient (2028). Furthermore, a fifth patient with an unknown diagnosis (2010) was also located in the same region, albeit on top of the 95% confidence ellipse region of the control patients (Figure 4a). The two patients suspected of Golgi homeostasis defects presented high quantities of the hypogalactosylated and hyposialylated glycans N5H3 (A3G0), as well as N4H4S1 (A2G1S1), among others. These glycans have previously been shown to be increased in ATP6V0A2-CDG by our group and others [16,21,29]. Nevertheless, the plasma N-glycans of the COG1-CDG patient evaluated presented a similar pattern, also displaying elevated concentrations of these two N-glycans, albeit not displaying alterations in glycans such as N5H4F1S1 (FA3G1S1), among others. The compositional similarity between the plasma profile of suspected CDG-II patients and previously diagnosed individuals with Golgi homeostasis defects prompted us to perform their molecular diagnostics, now directed at some putative Golgi disorders, by either using a gene panel and/or WES. The *ATP6V0A2* gene encodes the subunit a2 of the V-type proton ATPase protein, while the COG1 gene encodes the first subunit of the conserved oligomeric Golgi complex. Both are transport proteins involved in Golgi homeostasis, and similar N-glycan profiles for patients affected with ATP6V0A2-CDG and COG-CDG, as well as a normal N-glycome, have already been described [30].

A DNA sample from patient 2028 was extracted and subjected to gene panel analysis, and a novel, homozygous, pathogenic variant in the *ATP6V0A2* gene (c.721delA, p.Ile241Tyrfs*26) was found (Table 1). The electropherogram is depicted in Figure S3. No records in ClinVar or in the Franklin database were found for this variant; thus, it is novel. The DNA sample of patient 2010 was subjected to WES and, once again, a likely novel pathogenic variant (c.901C>T p.Gln301*) was found on the *ATP6V0A2* gene. This confirms that the proximity of cases in the PCA, given by their similar plasma N-glycan profile, is informative and can be used to infer a diagnosis; thus, it is useful in the interpretation of molecular sequencing data.

Interestingly, when evaluated altogether, the four ATP6V0A2-CDG patients and the COG1-CDG patient presented significantly increased quantities of hypogalactosylated and hyposialylated glycans, as anticipated, but also reduced levels of hybrid and mannose-rich N-glycans, as opposed to the MAN1B1-CDG patients. Reduced quantities of N2H6 (M6) and N2H9 (M9) were observed for these disorders, as well as N3H6S1 (A1G3S1). These differences help to explain why MAN1B1-CDG and Golgi homeostasis disorders are separated in the same principal components (PC2 and PC4), since they are affected by the same eigenvectors, but opposed in signal, occupying different quadrants.

### 2.5. Further Analyses of Principal Components and Model Limitations

Analysis of principal component 5 onwards did not display further diagnostic value and did not result in recognizable patterns regarding the classification of remaining undiagnosed and/or known CDG-II patients. Furthermore, despite explaining 12.72% of the data variance, PC3 also did not provide any useful information for CDG diagnostics, which means that the N-glycome variance related to the CDG-II subtypes was explained solely by using PC1, PC2, and PC4, comprising 45.35% of all the data variance.

Suspected CDG-II patients 2007, 2013, and 2016, who presented altered Tf-IEF profiles in previous analyses, were grouped with controls, suggesting a lack of alterations in their N-glycome. While the DNA samples for patients 2007 and 2016 were unfortunately unavailable, WES analysis of patient 2013 did not find any relevant variant, further suggesting that the patient’s condition is not CDG. Three other patients (2014, 2015, 2022) were outside the confidence region of controls for at least one pair of principal components but did not group with any available CDG-II subtype. Remarkably, the three of them are relatively close to each other in the first four components. Patients 2015 and 2022 were closest to the three control patients, who remained outside the confidence region for PC2 and PC4, which could be an indication of minor N-glycome alterations, possibly unrelated to CDG. Unfortunately, DNA samples from these patients were also not available for WES.

Notably, some of the cases of known CDG subtypes were not detected as different from the controls in our analysis. These include two SLC35A2-CDG patients, one TRAPPC11-CDG patient, and one ST3GAL3-CDG patient. These four patients were grouped with the controls in the components used for data analysis, meaning no alteration in their N-glycome was found. This is not unexpected, since alterations in plasma N-glycans of these subtypes have been elusive. A previous study with eight SLC35A2-CDG patients found no N-glycome alteration in skin fibroblasts, with minor O-glycosylation deficiency [31]. Conversely, increased levels of agalactosylated and monogalactosylated N-glycans have also been reported [32]. Our results did not reproduce these findings; instead, we observed a normal level of these glycans. The absence of alteration in the TRAPPC11-CDG patient differs from an earlier report that found reduced bi- and tri-antennary glycans (N4H5S2 and N5H6S3) and increased levels of biantennary glycans with an incomplete antenna (N3H5S1, N4H4S1, and N4H5S1) [33]. Alterations in the N-glycome of the ST3GAL3-CDG patient would likely be detected by using a sialic acid-specific derivatization methodology that allows the relative quantification of α-2,3 and α-2,6-linked structures [34,35]. This is because the ST3GAL3 enzyme specifically adds α-2,3 sialic acids to galactose motifs, which represent only a minor fraction of sialylated N-glycans [36].

## 3. Discussion

Sample preparation methods for glycomics are many and vary in N-glycan release, derivatization, chromatography, and mass spectrometry strategies [37,38]. In previous work, our group developed a methodology for the relative quantification of plasma N-glycans in CDG-II patients using stable-isotope labeling followed by mass spectrometry. Although this methodology was useful in the identification of alterations in specific N-glycan compositions for some patients, it demanded further data analysis to create an integrated platform for CDG diagnostics. Therefore, this experimental strategy was applied to 10 patients suspected of having CDG-II by comparing their N-glycome with 11 known CDG-II cases and 44 healthy controls using a multivariate statistical analysis. DNA samples from patients with altered N-glycome profiles, as detected in multivariate analysis of LC-MS/MS data, were submitted to gene sequencing, which confirmed the CDG-II diagnosis for four of them, demonstrating the effectiveness of combining glycomics and gene sequencing for CDG diagnosis.

**Figure 5:**
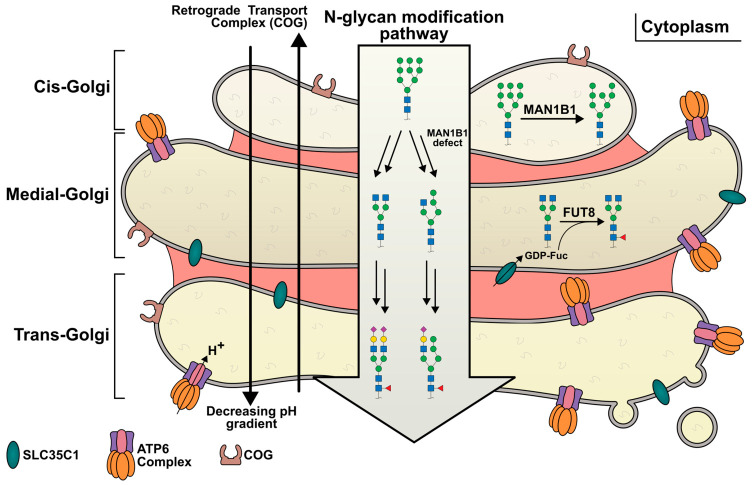
Schematic representation of the intracellular localization and function of the proteins affected by CDG subtypes exhibiting an altered glycome profile. The protein alpha-1,2-Mannosidase (MAN1B1), which has been observed in both the endoplasmic reticulum and the Golgi apparatus, removes one of the mannose residues from the immature oligomannosidic N-glycan. When it is defective, N-glycans with a hybrid structure accumulate. Fucosylation disorders can originate from defects in either the nucleotide-sugar transporter from the cytoplasm to the organelle (SLC35C1) or the fucosyltransferase 8 (FUT8) enzyme, which transfers the fucose residue to the N-glycan core. Defects in proteins involved in Golgi homeostasis (such as ATP6 and COG) influence glycosylation indirectly by disrupting the luminal pH gradient and retrograde transport required for normal organelle operation.

Principal component analysis of the relative quantities of plasma N-glycans revealed that control individuals have a converging profile, and that confidence ellipses can be traced to identify significant deviations from normality in a multidimensional space. Previously diagnosed individuals served as references, enabling the identification of typically altered glycan structures for each CDG-II subtype and consequently the inference of the putatively affected genes. Figure 5 illustrates the biological functions of the proteins affected in these specific CDG subtypes, contextualized within the compartmentalized N-glycosylation pathway [39]. The effect of the defect in proteins such as glycosidases, glycosyltransferases, and solute carriers is easier to predict due to their respective functions and will generally lead to the accumulation of or reduction in specific classes of glycans. On the other hand, proteins with regulatory functions that affect organelle homeostasis, such as ATP6V0A2 and COG1, lead to less obvious alterations. For instance, to fully understand why ATP6V0A2-CDG leads to the accumulation of under-sialylated N-glycans, one must consider its role in creating a pH gradient through the Golgi cisternae and how other proteins might be affected by this change in acidity. The alkalinization of the Golgi apparatus has been shown to cause mislocalization of sialyl- and glycosyltransferases, which directly correlated with the observed alterations in ATP6V0A2-CDG patients [40].

Therefore, this framework can be greatly enhanced by adding more patients with molecular diagnostics. The utility of the approach is exemplified by the identification of patients 2011 and 2012 as novel MAN1B1-CDG cases, hinted by glycan analysis and confirmed by whole exome sequencing. The two novel ATP6V0A2-CDG cases (2010 and 2028) also highlight the utility of this platform. The similarity between glycan profiles is not given by subjective analysis or by user experience, but by a multidimensional similarity score based on qualitative and quantitative alterations in glycan compositions. This constitutes a helpful tool for experienced and inexperienced clinicians and might be particularly relevant for some CDG-II subtypes, which present overlapping alterations, such as Golgi trafficking disorders [41]. As soon as novel patients are introduced to the platform, we will be able to evaluate the exact potential of the current applied methodology in discerning between subtypes not yet present in our cohort. We expect that these subtypes will form clusters, possibly affecting different principal components than the subtypes already analyzed, in a way that the results also point to them specifically.

Out of the ten putative CDG-II patients, four were confirmed as CDG-II, which consists of a relatively low rate of diagnosis with the present approach. This is in contrast to what is reported for CDG-I patients using a similar glycomics–genomics platform in the literature, which led to the molecular diagnosis of 78% of cases [12]. A few considerations should be made. The criteria for inclusion in the suspected CDG-II patient cohort in the present study were an altered Tf IEF profile and suggestive clinical manifestations. However, both features lack precision as indicators of CDG, especially for CDG-II. The Tf IEF profile observed in CDG-II cannot be clearly distinguished from secondary glycosylation alterations [21] and is not as characteristic as that observed for CDG-I [3]. The typical Tf IEF profile of CDG-II involves quantitative alterations in trisialo-, tetrasialo-, and pentasialotransferrin isoforms, which are less specific than those seen in CDG-I, which presents increased asialo- and disialotransferrin bands. The capacity to detect small deviations from normality is what makes glycomics useful in clinical chemistry [42]. A previous study found that 60% of the 207 patients screened by the NIH Undiagnosed Disease Program had at least one fluid (plasma or urine) with altered levels of N- and O-glycans [43]. This indicates that glycan alterations are common in patients suspected of having rare genetic disorders in general and are by no means exclusive to CDGs. Interestingly, only 7.2% (15 out of 207) of these patients had CDG as a primary cause, indicating that most of them have secondary glycosylation defects [43]. Finally, some other diagnostic limitations might have been introduced by the characteristics of some CDG subtypes. For example, normalization of the Tf IEF profile after a few years of age has been observed for SLC35A2-CDG patients [44], and this phenomenon could also be occurring with the N-glycome. Considering the patients studied in this work were approximately 10 years old when samples were collected, as well as the fact that SLC35A2-CDG is an X-linked disease that can occur as mosaicism, they were likely past the diagnostic window of the disease, impairing their diagnosis by this method.

The approach presented herein was successful in identifying glycosylation alterations that are directly related to the underlying glycobiology of some CDG subtypes. Indeed, subtypes that have a common etiology, like deficient fucosylation, accumulation of hybrid glycans, or production of hypogalactosylated and/or hyposialylated glycan structures, were grouped in the principal components, despite the fact that no information on group memberships was provided to the model. This constitutes an interesting system of reference for the understanding of pathologies that result in N-glycan alterations that have not been used to build the model, such as novel CDG subtypes [45], cancer [46], or even some degenerative diseases [47]. In this sense, the present platform is complementary to other methods of CDG diagnosis, based on either intact transferrin analysis by mass spectrometry [14] or by glycoproteomics [18].

## 4. Materials and Methods

Unless otherwise stated, all chemicals and reagents were purchased from Sigma-Aldrich (Merck KGaA, Darmstadt, Germany).

### 4.1. Samples

Forty-four individuals from the Rede Sarah de Hospitais de Reabilitação and the Hospital Universitário de Brasília were selected as the control group based on the following criteria: age 0–13 years and the absence of symptoms compatible with CDG. The CDG group included 10 patients with a confirmed molecular diagnosis of CDG-II. A list of patients, their diagnostics, and the detected variants is given in Table 2. Finally, 10 patients with CDG-compatible phenotype, but without a confirmed diagnosis, were selected for the ‘no diagnosis’ (Undx) group.

### 4.2. Relative Quantification of N-Glycans

Relative N-glycan quantification was conducted on a cohort comprising 44 controls, 11 CDG-II patients, and 10 individuals with suspected CDG-II using the method previously described, with adaptations [16]. Briefly, the plasma of the control individuals was pooled to create the “control pool”, which was subsequently prepared using the same protocol used on the patient samples. Aliquots of 40 µL of plasma from the control, CDG-II, and undiagnosed patients were used for the analysis. Each sample was denatured (0.6 M tris and 6 M guanidine buffer), reduced (500 mM DTT), alkylated (3 M iodoacetamide), followed by trypsin digestion (24 h at 37 °C) and N-glycans release using PNGase F (17 h at 37 °C).

Released glycans were purified using C18-E solid phase extraction (SPE) (Phenomenex, Torrance, CA, USA) and derivatized with iodomethane (patient samples) or deuterated iodomethane (control pool). Then, each patient sample was mixed in a 2:1 ratio with the control pool, prior to final purification by C18-E SPE. These samples containing both I-CH_3_ and I-CD_3_-derivatized N-glycans were separated by liquid chromatography using an Agilent 1260 Infinity II chromatograph equipped with a Kinetex Core–Shell C18 column (50 × 4.6 mm, 2.6 µm; Phenomenex). Mobile phases A and B consisted of MS-grade water and methanol, respectively, both containing 0.1% (*v*/*v*) formic acid and 80 µM NaOH. The column was connected to a TripleTOF 5600+ mass spectrometer (AB Sciex, Marlborough, MA, USA) operating in the positive mode using information-dependent acquisition (top 6 ions with 1–4 charges, *m*/*z* greater than 600 and at least 800 cps intensity).

### 4.3. Data Analysis

N-Glycans were annotated based on previous experience from the group, as well as an in-house script that searched for possible compositions in the GlyGen database [54]. Manual analysis of the mass spectra of all glycans was performed to confirm annotation with the aid of GlycoWorkbench 2.1 [55]. Extracted ion chromatograms and respective peak areas were obtained using the MultiQuant software version 3.0.3 (AB Sciex). The quantification of each identified glycan was performed using the signal of the deuterated glycan or of a similar glycan (for glycans with low abundance in controls) as an internal standard. Each glycan ratio was normalized by the sample-wide ratio of summed reference glycans to summed reference internal standards to minimize inter-sample variance, followed by a log transformation to correct the skewness inherent to ratio-based data.

Statistical analysis was done using the R statistical language version 4.5.2. The standardized glycan ratios were subjected to principal component analysis (PCA).

### 4.4. DNA Analysis

Molecular diagnostics of putative CDG-II-affected patients were performed using a gene panel or by whole-exome sequencing (WES). The next-generation sequencing panel consisted of a total of 18 genes (*ATP6V0A2*, *B4GALT1*, *CCDC115*, *COG1*, *COG4*, *COG5*, *COG6*, *COG7*, *COG8*, *MGAT2*, *MOGS*, *PGM1*, *SLC35A1*, *SLC35A2*, *SLC35C1*, *SLC39A8*, *TMEM165*, *TMEM199*) associated with CDG-II. Enrichment was performed with a Custom SureSelect QXT Panel (Agilent Technologies, Inc., Santa Clara, CA, USA) prior to NGS on an MiSeq instrument (Illumina, San Diego, CA, USA). The sequences were analyzed in the SureCall software (v. 4.1.2.11) (Agilent Technologies, Inc., Santa Clara, CA, USA) and variants were classified according to the literature [56]. All coding exons of the RefSeq transcripts of the genes and 20 base pairs flanking introns were targeted. Over 99% of the coding exons of all genes in the panel were sequenced to a read depth of 30× or greater in almost all cases.

The *MAN1B1* gene was evaluated by Sanger sequencing. A total of 13 amplicons were used to analyze the 13 coding exons, and 20 base pairs of the flanking introns were targeted. Similarly, the ATP6V0A2 gene was Sanger sequenced (20 exons, 20 base pairs of the flanking introns). Hg19 was the reference genome applied.

WES was performed using the NovaSeqX (Illumina) sequencer and Twist Bioscience exome capture kit (South San Francisco, CA, USA) at a sequencing facility (Genesis Genomics, São Paulo, Brazil). Data were aligned to the hg19/GRCh37 assembly of the human genome reference, annotated, and analyzed using a commercially available interpretation platform (Franklin, Qiagen, Germantown, MD, USA).

The variants were classified according to the American College of Medical Genetics (ACMG). For the variant analysis, public databases were used, including Exome Aggregation Database (ExAC), Arquivo Brasileiro Online de Mutações (ABraOM), Genome Aggregation Database (gnomAD), 1000 Genomes, and the Single Nucleotide Polymorphism Database (dbSNP), Clinical Variant Database (ClinVar), and OMIM. In silico tools such as PolyPhen-2 (https://genetics.bwh.harvard.edu/pph2/, accessed on 3 March 2026), SIFT (https://siftdna.org/, accessed on 3 March 2026), CADD 1.7 build GRCh37 / hg19, and MutationTaster2021 (https://www.genecascade.org/MutationTaster2021/, accessed on 3 March 2026) were used to predict the functional impact of the variants.

## 5. Conclusions

We demonstrate how multivariate statistics might be used to ease the interpretation of glycomics data in putative CDG-II patients. This strategy is interesting as a complement to Tf IEF and WES in the diagnostics of novel cases for the following reasons: 1. The interpretation of glycan alterations is often challenging and dependent on the clinician’s experience. Therefore, this approach allows even less experienced clinicians to recognize similar N-glycosylation patterns among patients, speeding up the diagnostics. 2. If more data is acquired with novel controls and CDG-II individuals with molecular diagnostics using the same methodology, the predictive power of the data analysis should be increased. 3. It has the potential to be used for therapy monitoring, since an effective treatment is expected to display the transition from an altered glycosylation pattern to normality over the course of the treatment. 4. Spatial proximity in the PCA plots between N-glycan profiles can indicate similar altered phenotypes even if not caused by the same gene, thus aiding the advancement in the correlation between genotype and phenotype. Consequently, this approach facilitates the identification and functional characterization of novel CDG cases involving genetic variants not currently associated with the disease or cataloged within OMIM. It is our understanding that this (or a similar) methodology, encompassing more known CDG-II subtypes, can be applied to patients with clinical manifestations compatible with glycosylation disorders as an approach to direct genetic sequencing, thus impacting the time to reach diagnosis.

## Figures and Tables

**Table 1:** Gene sequencing of suspected CDG-II patients.

Patient	Method	Gene	Variant	ClinVar	Zygosity	ACMG Classification
2010	WES	*ATP6V0A2*	c.901C>T chr12:124221681C>T p.Gln301*	Novel	Homozygous	Pathogenic PVS1, PM2, PM3
2011	WES	*MAN1B1*	c.1735dup chr9:140001865A>AC p.Gln579Profs*75	VCV001207945.4	Homozygous	Pathogenic PM3, PVS1, PM2
2012	WES	*MAN1B1*	c.1735dup chr9:140001865A>AC p.Gln579Profs*75	VCV001207945.4	Homozygous	Pathogenic PM3, PVS1, PM2
2028	Gene panel	*ATP6V0A2*	c.721delA chr12:123733993-123734003delA p.Ile241Tyrfs*26	Novel	Homozygous	Pathogenic PVS1, PM2, PM3

*ATP6V0A2*: ATPase H^+^ transporting V0 subunit a2, and *MAN1B1*: mannosidase alpha class 1B member 1.

**Table 2:** Patients with a confirmed CDG diagnosis.

Patient	Gene	Variant	Zygosity	ACMG Classification	Variant ClinVar/Reference
2001	*COG1*	c.2665_2666insC; p.Arg889Profs*12	Homozygous	Pathogenic PM3, PVS1, PM2	N.F./N.F.
2002	*SLC35A2*	c.1A>T; p.Met1Leu	Heterozygous	Pathogenic PS1,PVS1, PM2, PP5	VCV001686209.2/N.F.
2003	*SLC35A2*	c.193_204del; p.Tyr65_Leu68	Heterozygous	VUS PM4, PM2	N.F./[48]
2004	*TRAPPC11*	c.913_914delAA; p.Lys305Aspfs*9 c.2938G>A; p.Gly980Arg	Compound Heterozygous	Pathogenic PM3, PP1, PS3, PM2, PP3 Pathogenic PVS1, PM2, PP5	VCV002141268.4/N.F. VCV000060510.20/[49]
2006	*FUT8*	c.796T>C; p.Cys266Arg c.55G>C; p.Gly19Arg	Compound Heterozygous	VUS PM2, PP3, PM3(support) VUS PM2, PM3 (support)	VCV001690905.2/N.F. VCV001690906.2/N.F.
2017	*MAN1B1*	c.1225T>C; p.Ser409Pro c.1282delA; p.Ile428Serfs*43	Compound Heterozygous	Likely Pathogenic PM2, PM3, PP3, PP5 Likely Pathogenic PVS1, PM2	VCV000983290.1/[50] N.F./[27]
2020	*ATP6V0A2*	c.187C>T; p.Arg63Ter	Homozygous	Pathogenic PVS1, PM2, PP5	VCV000000845.17/[51]
2021	*ATP6V0A2*	c.187C>T; p.Arg63Ter	Homozygous	Pathogenic PVS1, PM2, PP5	VCV000000845.17/[51]
2027	*MAN1B1*	c.1607G>T; p.Gly536Val	Homozygous	VUS PM2, PP3, PM3 (support)	N.F./[16]
2023	*ST3GAL3*	c.1060C>T p.Arg354Ter	Homozygous	Pathogenic PVS1, PM2, PM3	N.F./N.F.
2030	*SLC35C1*	c.439C>T; p.Arg147Cys	Homozygous	Likely Pathogenic PM3, PS3, PM2, PP3	VCV000004739.6/[52,53]

*COG1*: component of oligomeric golgi complex 1; *SLC35A2*: solute carrier family 35 member A2; *TRAPPC11*: trafficking protein particle complex subunit 11; *FUT8*: fucosyltransferase 8; *MAN1B1*: mannosidase alpha class 1B member 1; *ATP6V0A2*: ATPase H^+^ transporting V0 subunit a2; *ST3GAL3*: ST3 beta-galactoside alpha-2,3-sialyltransferase 3; *SLC35C1*: solute carrier family 35 member C1; N.F.: not found.

## Data Availability

The raw LC-MS data files generated during this study have been deposited in the GlycoPOST database under the dataset identifier GPST000712.

## Supplementary Materials

The following supporting information can be downloaded at: https://www.mdpi.com/article/10.3390/ijms27146309/s1.

## Abbreviations

The following abbreviations are used in this manuscript:

ABraOM	Arquivo Brasileiro Online de Mutações
ACMG	American College of Medical Genetics
CDG	Congenital Disorders of Glycosylation
CDG-II	Congenital Disorders of Glycosylation Type II
CE	CapillaRy Electrophoresis
ClinVar	Clinical Variant Database
dbSNP	The Single Nucleotide Polymorphism Database
ER	Endoplasmic Reticulum
ExAC	Exome Aggregation Database
GA	Golgi Apparatus
GDP	Guanosine Diphosphate
gnomAD	Genome Aggregation Database
HPLC	High-Performance Liquid Chromatography
IDA	Information-Dependent Acquisition
LC-MS	Liquid Chromatography–Mass Spectrometry
MS	Mass Spectrometry
PCA	Principal Component Analysis
SIL	Stable Isotopic Labeling
SPE	Solid Phase Extraction
Tf-IEF	Isoelectric Focusing of Transferrin
WES	Whole Exome Sequencing
WGS	Whole Genome Sequencing
XIC	Extracted Ion Chromatogram
