# Supplementary material for: Diagnosis of Congenital Disorders of Glycosylation Type II Subtypes Through Comprehensive N-Glycan Profiling by Mass Spectrometry
Source: Int J Mol Sci. 2026 Jul 15;27(14):6309. doi: 10.3390/ijms27146309 (PMC13411261; doi:10.3390/ijms27146309)
Supplement: Supplementary file 1 [file ijms-27-06309-s001.zip › Supplementary Figures.pdf]

Diagnosis of congenital disorders of glycosylation type II subtypes through comprehensive N-glycan profiling by mass spectrometry

Alan R. Mól[1], Nilza do C. Fontes[2], Savana C. L. Santos[3], Cynthia Costa e Silva[3], Gerson da S. Carvalho[4], Bruno J. C. B. Lima[5], Walquíria D. de Mello[6], Daniel R. de Carvalho[2], Eder A. Barbosa[1], Dirk J. Lefeber[7], Juliana F. Mazzeu[8], Jaime M. Brum[1], Guilherme D. Brand[1]*

[1] Laboratório de Síntese e Análise de Biomoléculas – LSAB, Instituto de Química, Universidade de Brasília, Brasília, Brasil.

[2] Laboratório de Genética Bioquímica, Rede Sarah de Hospitais de Reabilitação, Brasília, Brasil.

[3] Laboratório de Biologia Molecular, Rede Sarah de Hospitais de Reabilitação, Brasília, Brasil.

[4] Hospital de Apoio de Brasília, Secretaria de Saúde do Distrito Federal, Brasília, Brasil

[5] Hospital Universitário Júlio Muller, Faculdade de Medicina, Universidade Federal de Mato Grosso, Cuiabá, Brasil

[6] Clínica de Pediatria do Hospital Infantil João Paulo II, Fundação Hospitalar do Estado de Minas Gerais, Belo Horizonte, Brasil.

[7] Translational Metabolic Laboratory, Department of Human Genetics, Donders Center for Brain, Cognition, and Behavior, Radboud University Medical Center, Nijmegen, the Netherlands.

[8] Laboratório de Genética Clínica, Faculdade de Medicina, Universidade de Brasília, Brasília, Brasil.

* Corresponding author:

E-mail address: gdbrand@unb.br (Guilherme D. Brand)

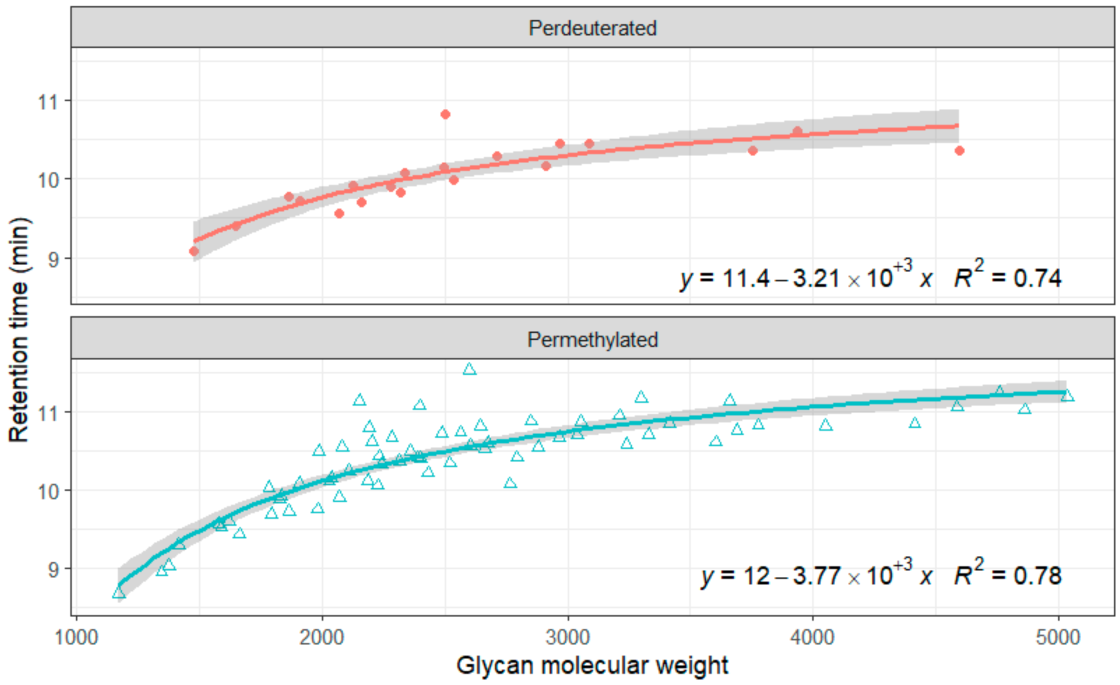

Supplementary Figure S1 Derivatized glycans showed high correlation between their retention time and molecular weight. Permethylated and perdeuterated glycans were fit separately because labeled glycans usually elute before their methylated counterparts, despite the higher molecular weight

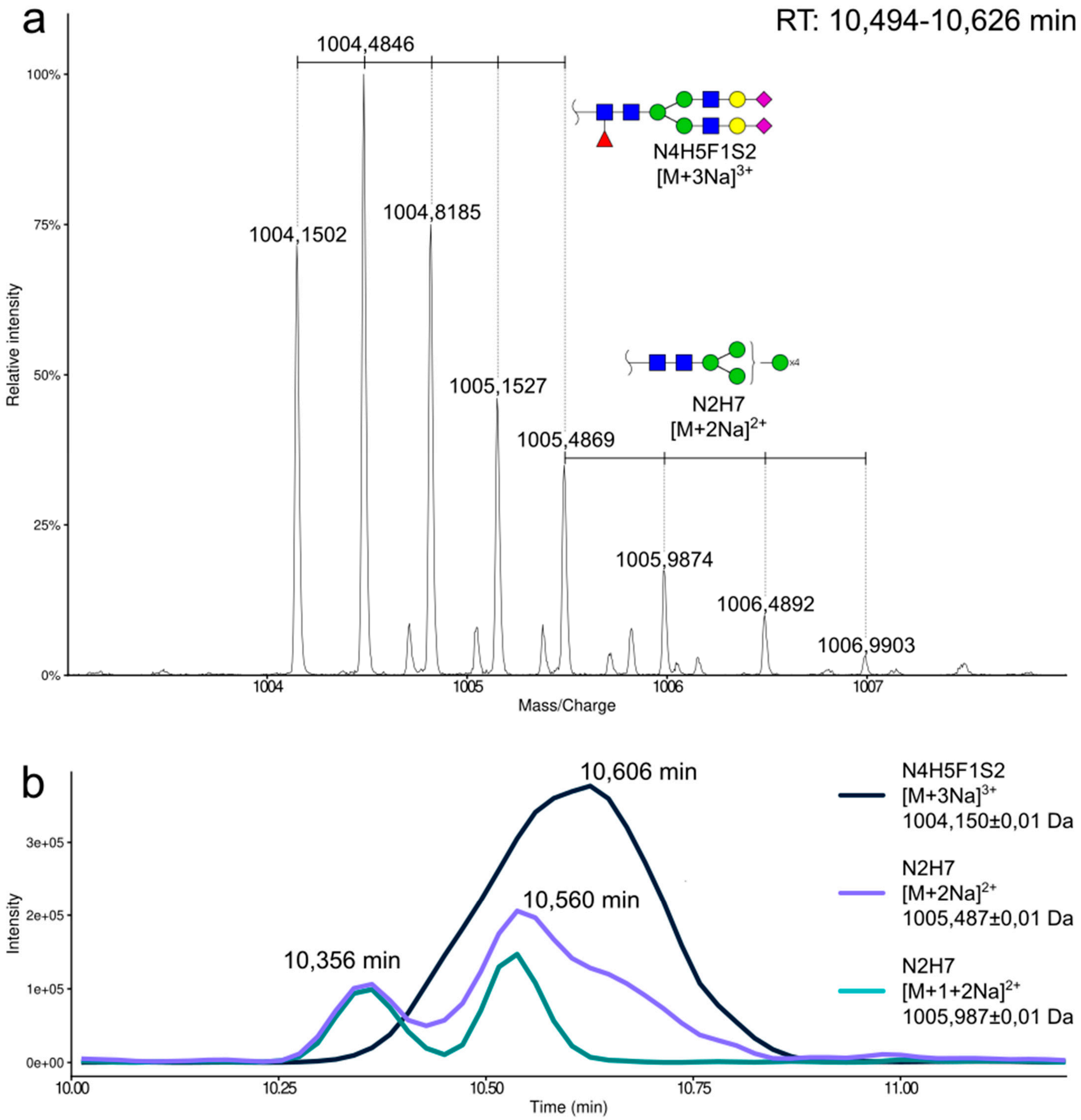

Supplementary Figure S2 Different glycan compositions can have overlapping mass peaks. (**a**) The fucosylated biantennary glycan N4H5F1S2 is very abundant, and its $[M+4+3Na]^{3+}$ isotopologue has a mass/charge ratio very close to N2H7's $[M+2Na]^{2+}$ (1005.4837 and 1005.4854). (**b**) Extracted ion chromatograms of the discussed ions, showing partial coelution between N4H5F1S2 and N2H7. When *m/z* 1005.4834 is extracted, the peak is affected by both glycan structures. Extracting N2H7's $[M+1+2Na]^{2+}$ this can be prevented and the peak shape corresponds only to this structure

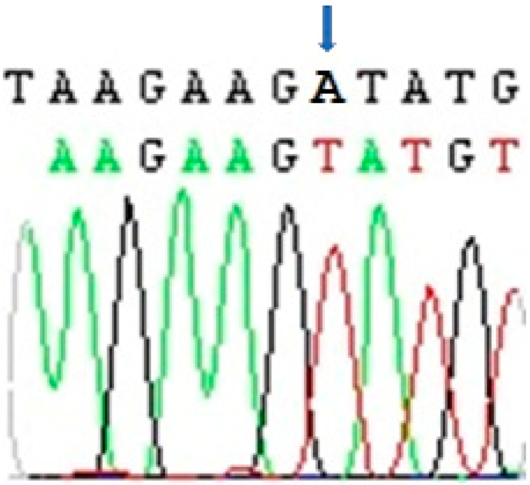

Supplementary Figure S3 Sanger sequencing of patient 2028. Above: Normal sequence. Below: Frameshift generated by deletion of an A (arrow) and the resulting sequence.

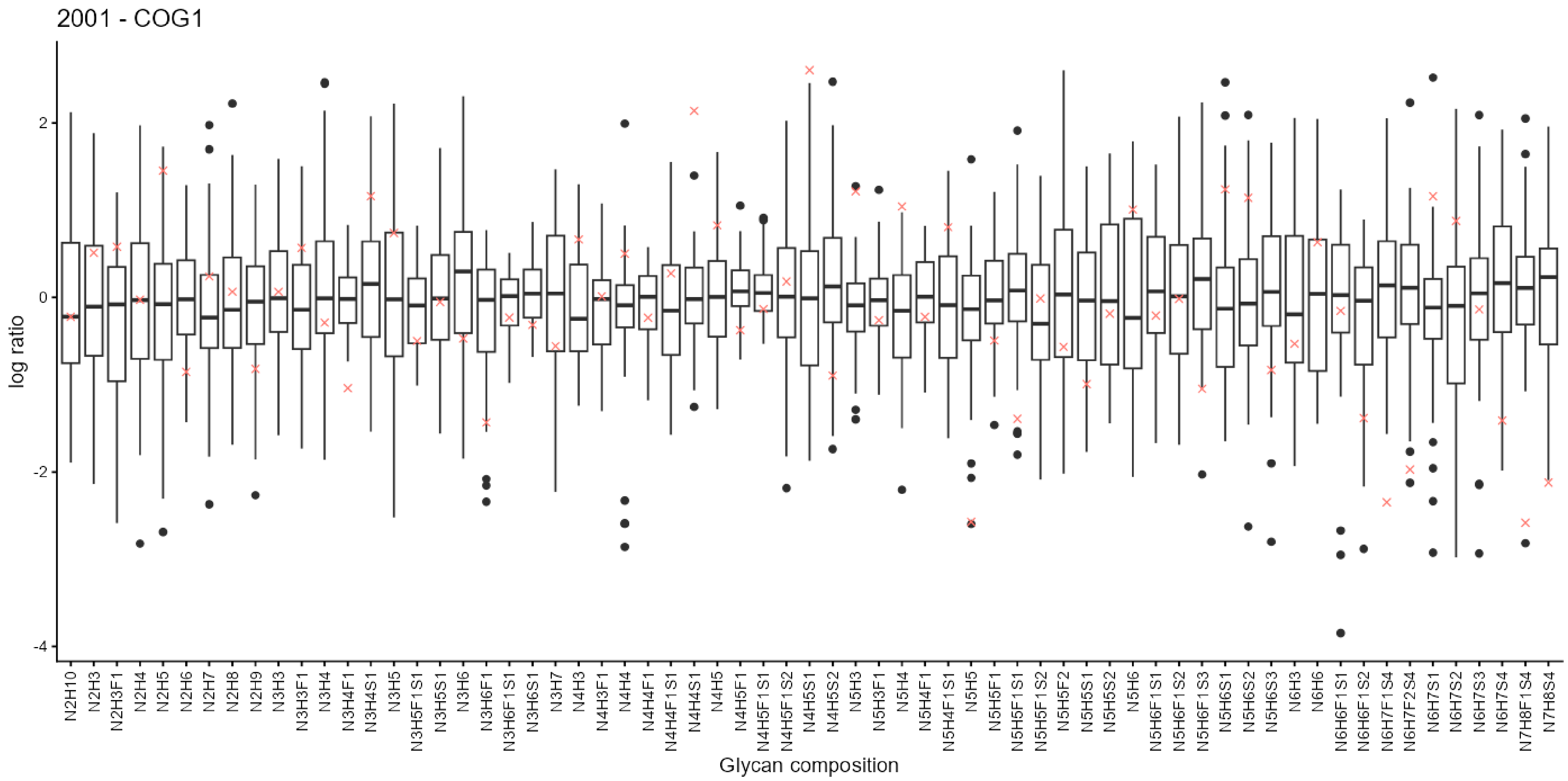

Supplementary Figure S4 Relative quantification of *N*-glycans for patient 2001. Red crosses represent the $\log_{10}$ abundance ratios of N-glycans from the patient relative to the control pool. Black circles indicate individual control values identified as outliers

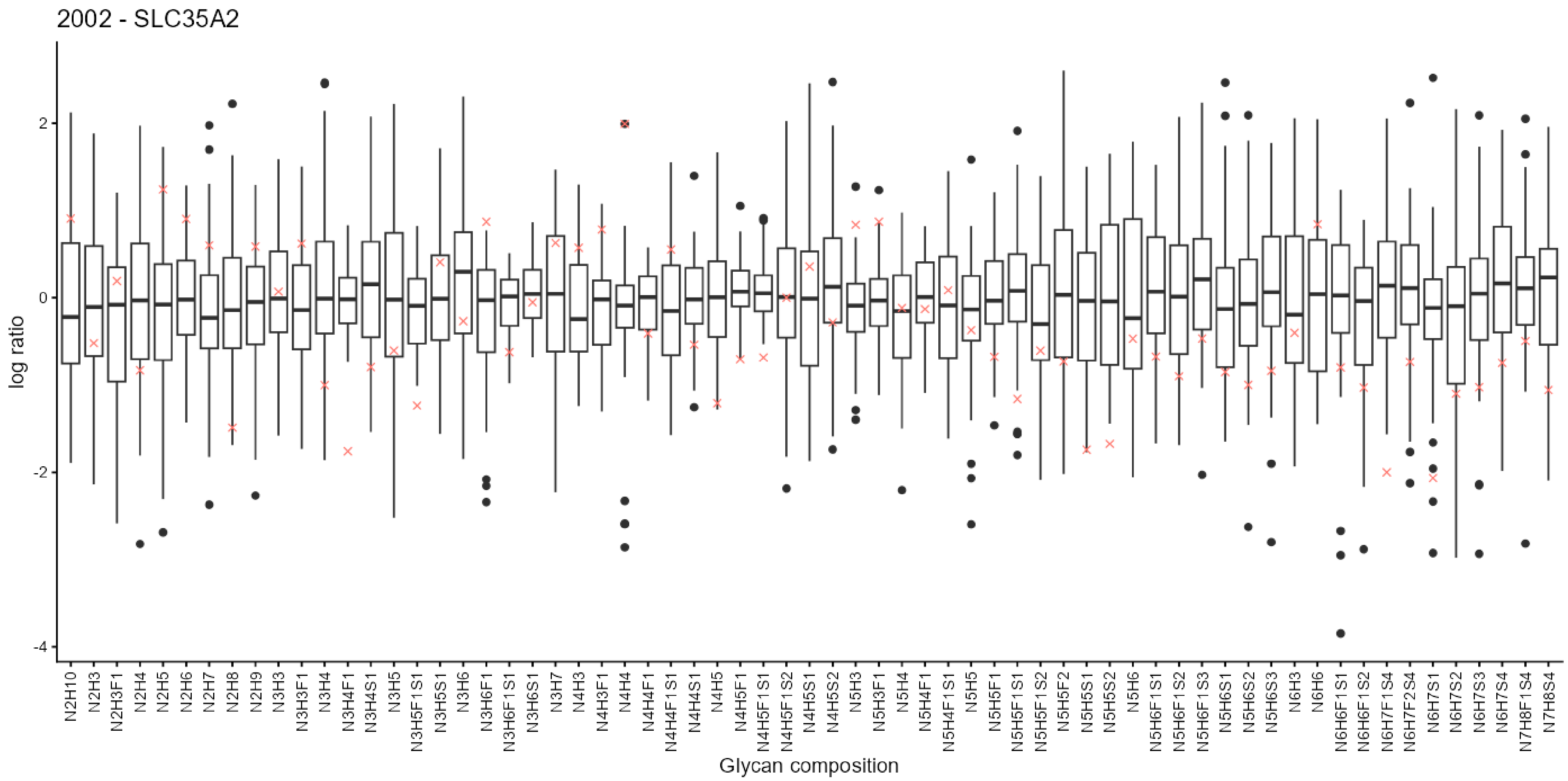

Supplementary Figure S5 Relative quantification of *N*-glycans for patient 2002. Red crosses represent the $\log_{10}$ abundance ratios of N-glycans from the patient relative to the control pool. Black circles indicate individual control values identified as outliers

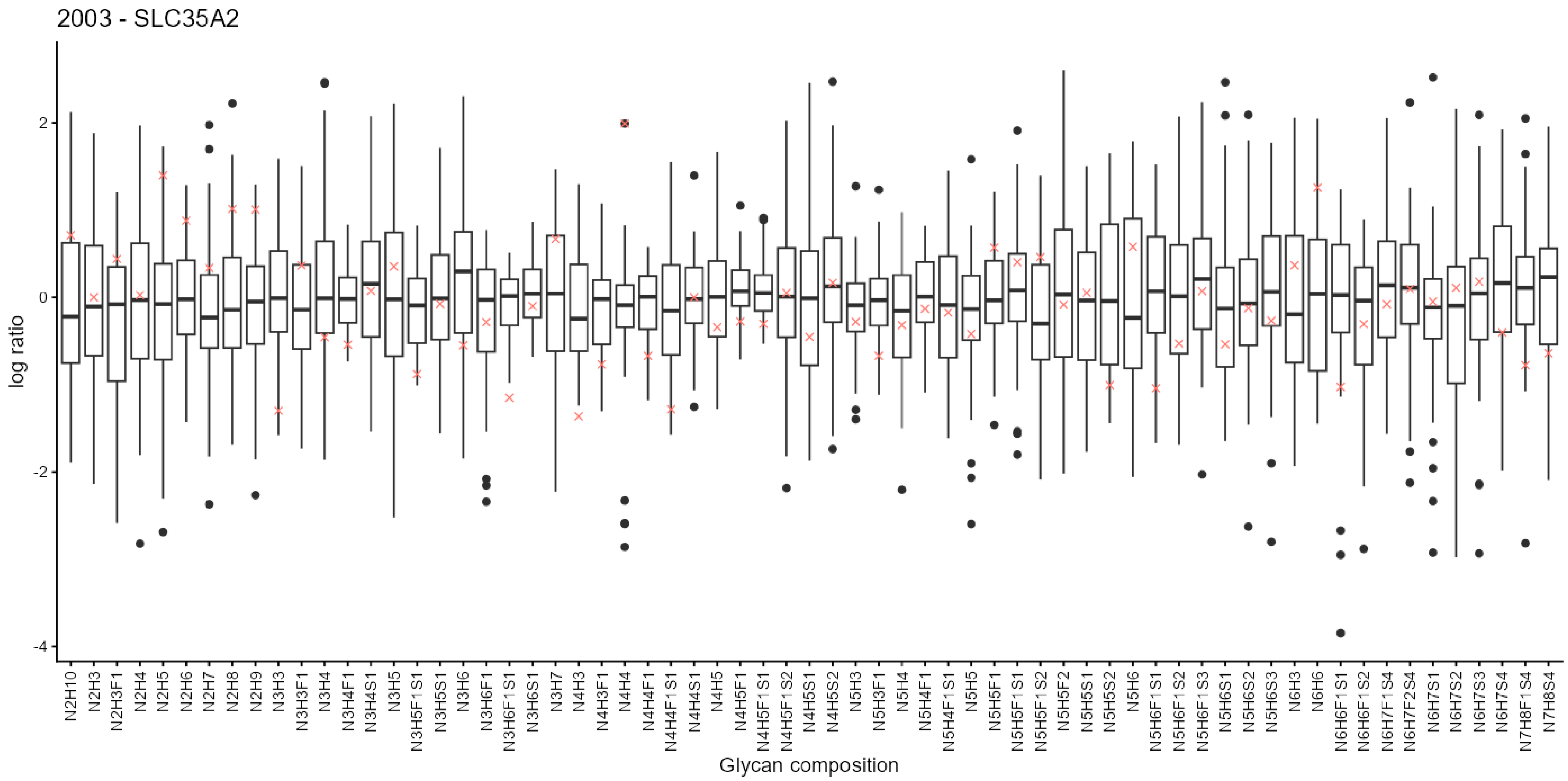

Supplementary Figure S6 Relative quantification of *N*-glycans for patient 2003. Red crosses represent the log_{10} abundance ratios of N-glycans from the patient relative to the control pool. Black circles indicate individual control values identified as outliers

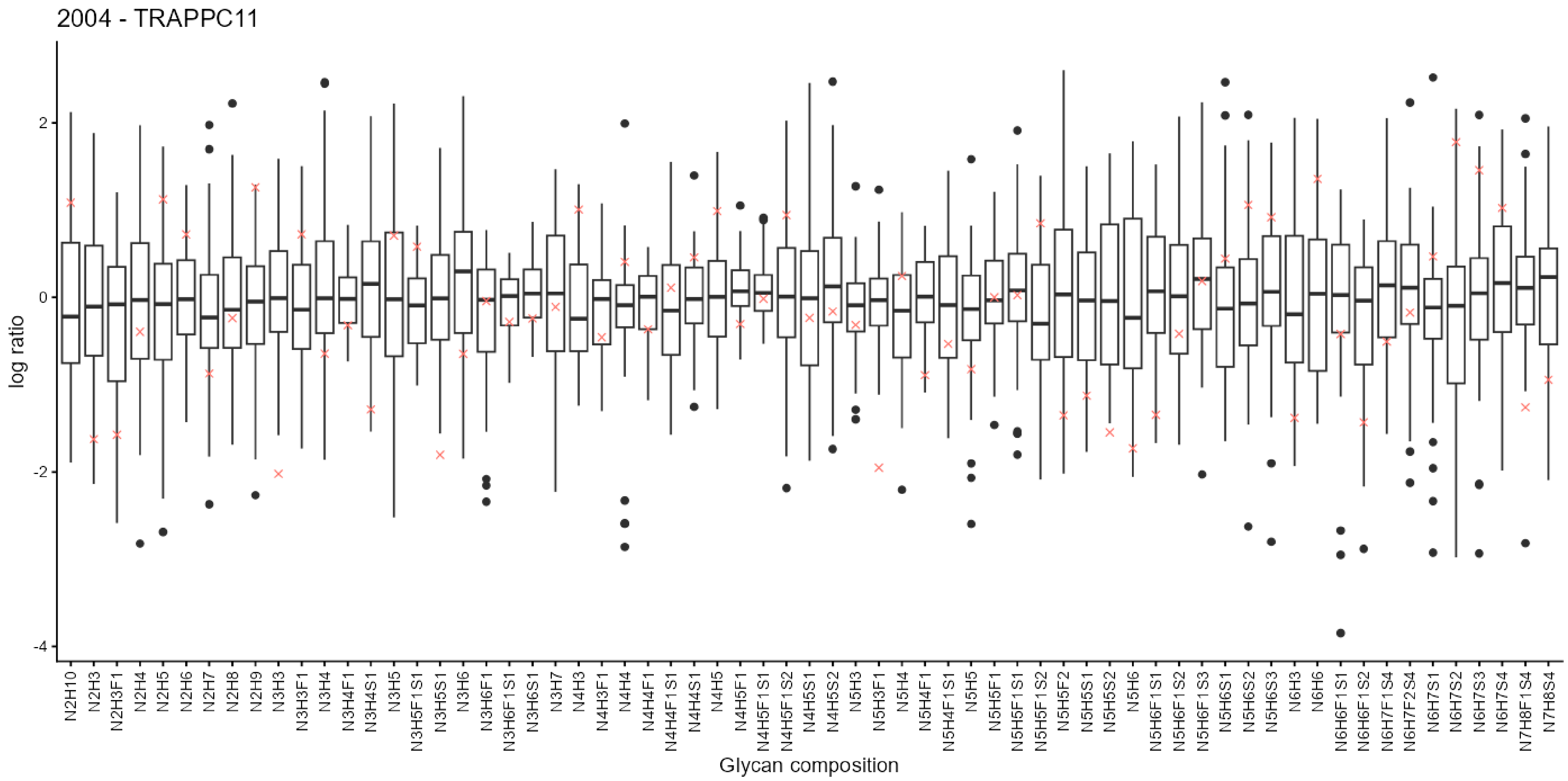

Supplementary Figure S7 Relative quantification of *N*-glycans for patient 2004. Red crosses represent the $\log_{10}$ abundance ratios of N-glycans from the patient relative to the control pool. Black circles indicate individual control values identified as outliers

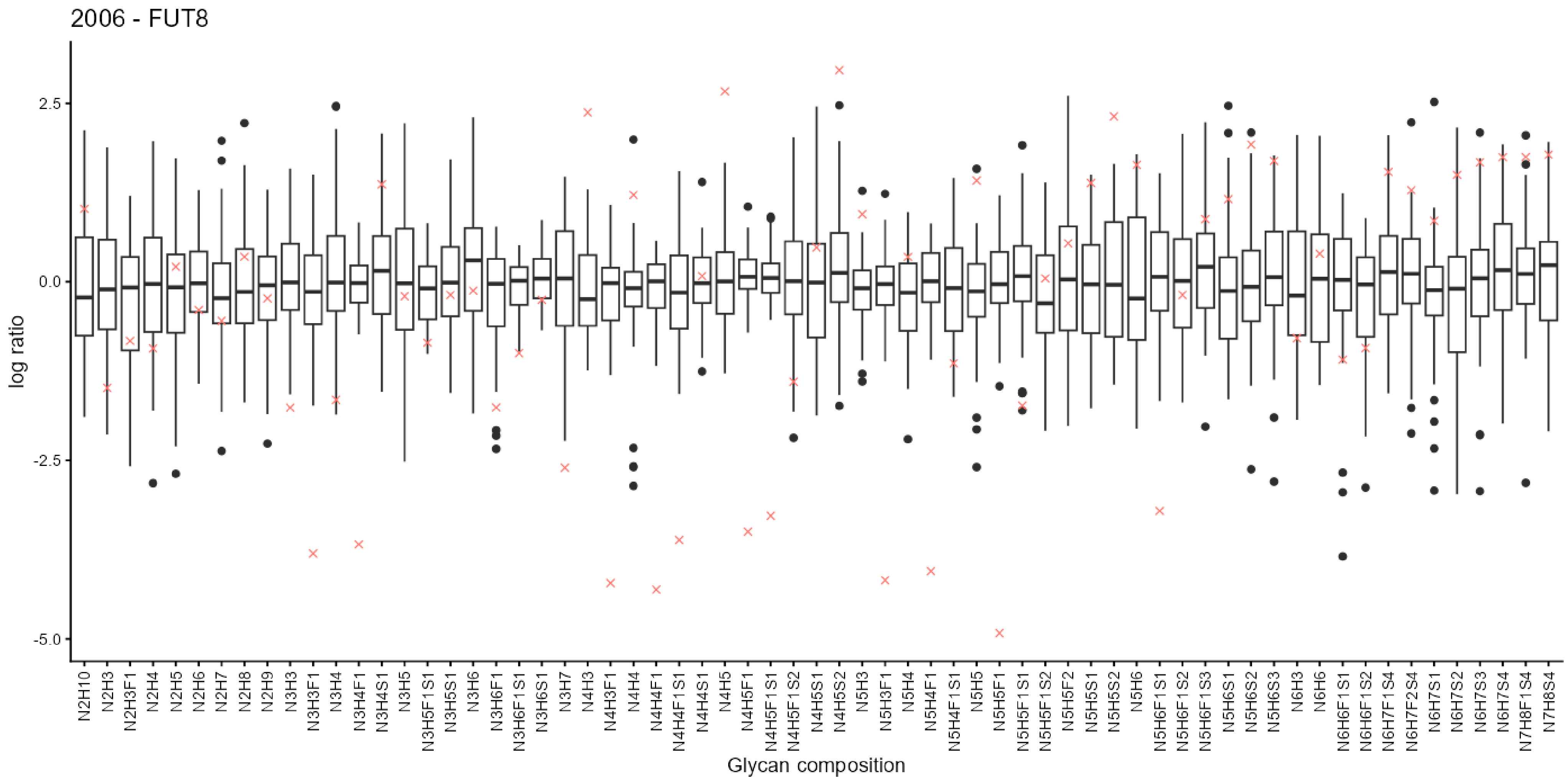

Supplementary Figure S8 Relative quantification of *N*-glycans for patient 2006. Red crosses represent the $\log_{10}$ abundance ratios of N-glycans from the patient relative to the control pool. Black circles indicate individual control values identified as outliers

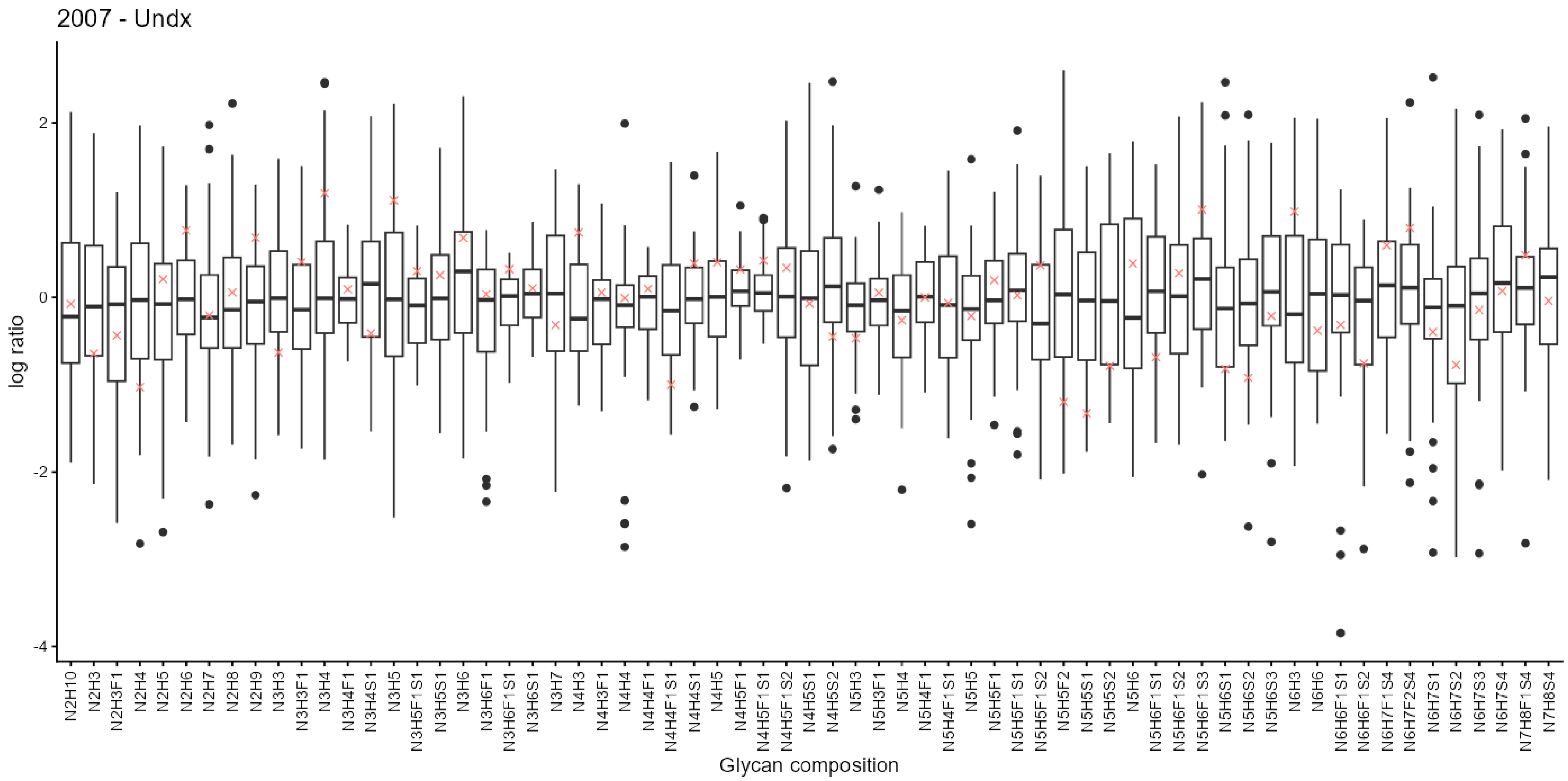

Supplementary Figure S9 Relative quantification of *N*-glycans for patient 2007. Red crosses represent the log_{10} abundance ratios of N-glycans from the patient relative to the control pool. Black circles indicate individual control values identified as outliers

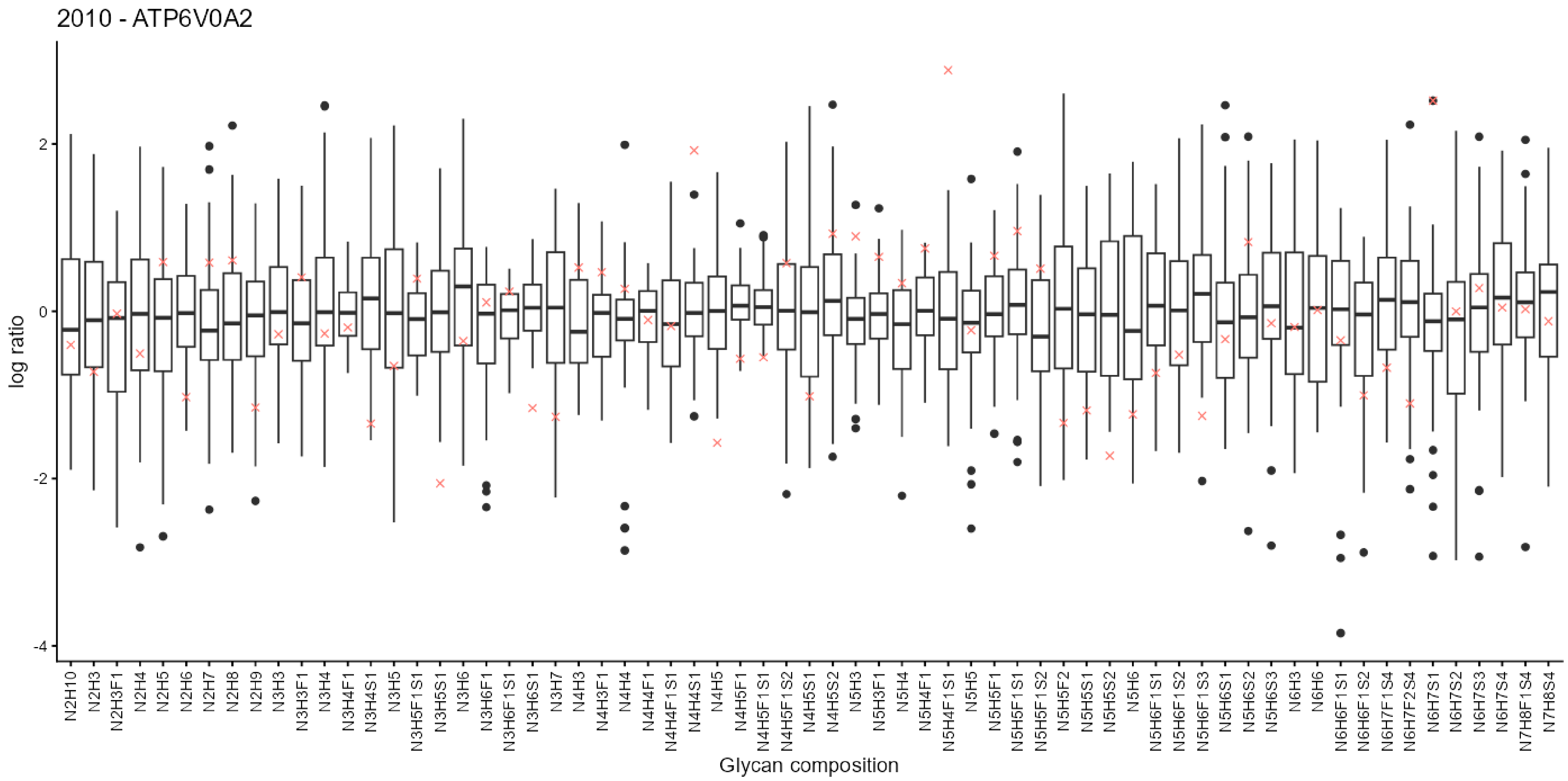

Supplementary Figure S10 Relative quantification of *N*-glycans for patient 2010. Red crosses represent the log_{10} abundance ratios of N-glycans from the patient relative to the control pool. Black circles indicate individual control values identified as outliers

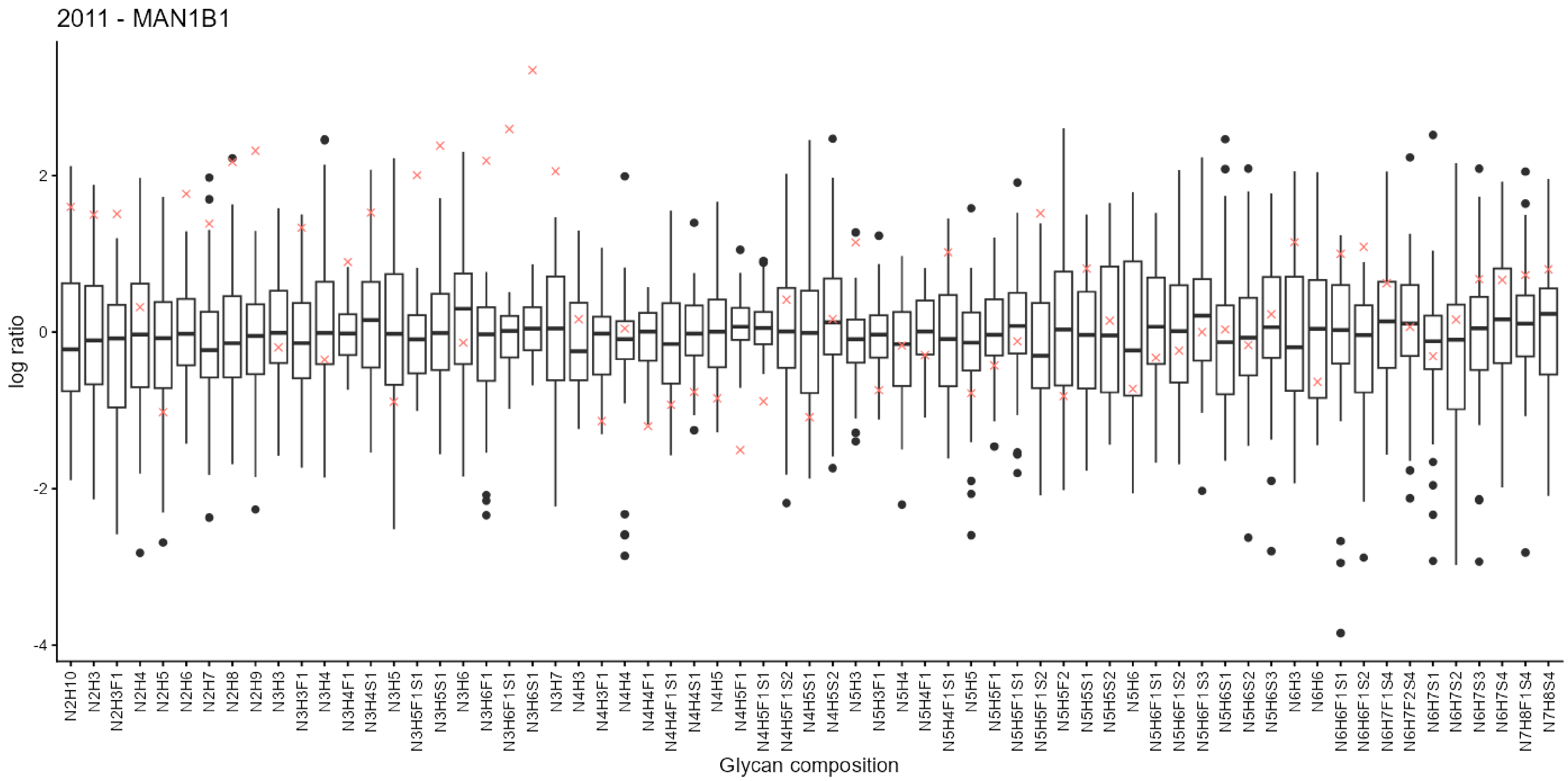

Supplementary Figure S11 Relative quantification of *N*-glycans for patient 2011. Red crosses represent the $\log_{10}$ abundance ratios of N-glycans from the patient relative to the control pool. Black circles indicate individual control values identified as outliers

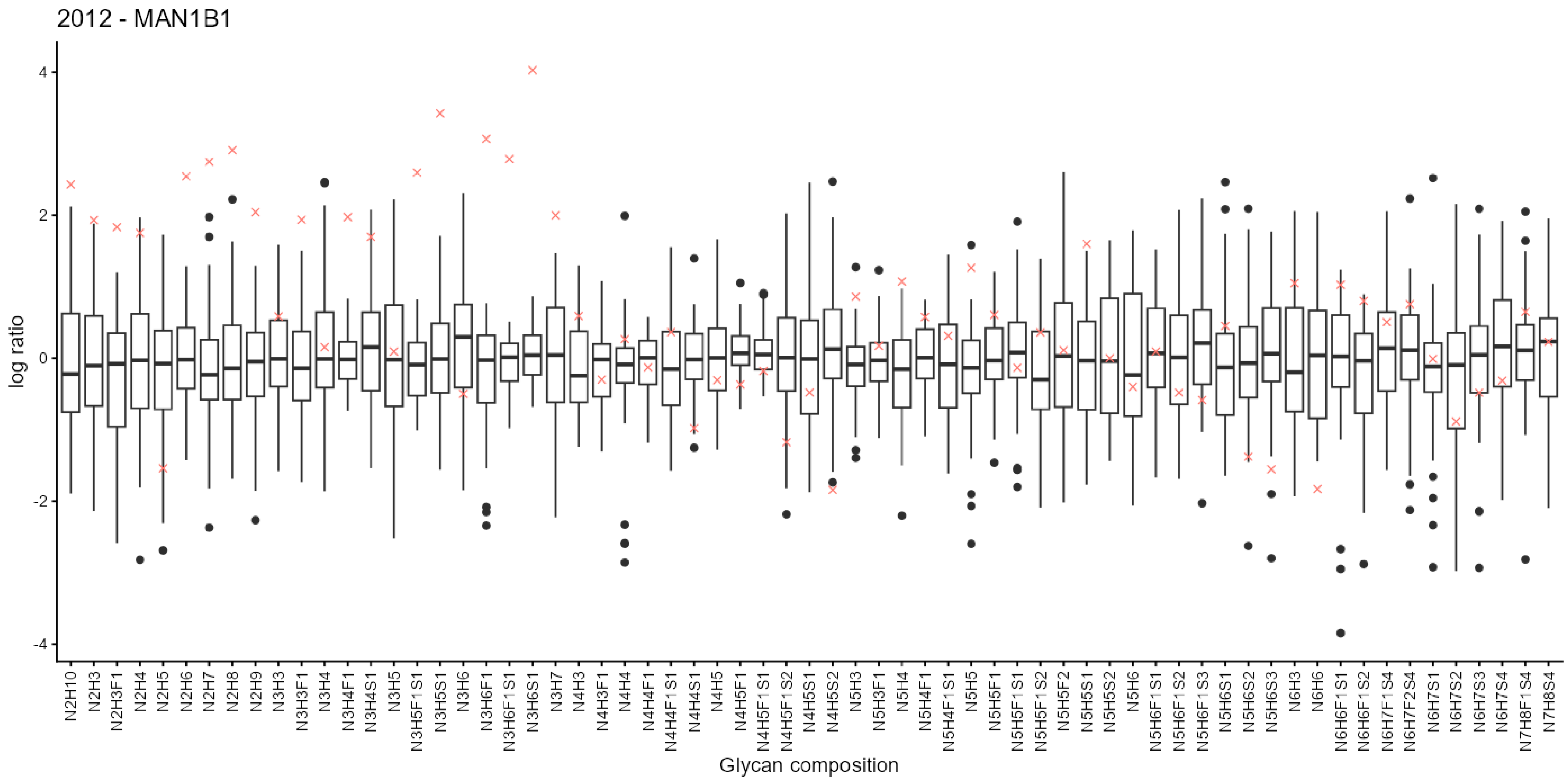

Supplementary Figure S12 Relative quantification of *N*-glycans for patient 2012. Red crosses represent the $\log_{10}$ abundance ratios of N-glycans from the patient relative to the control pool. Black circles indicate individual control values identified as outliers

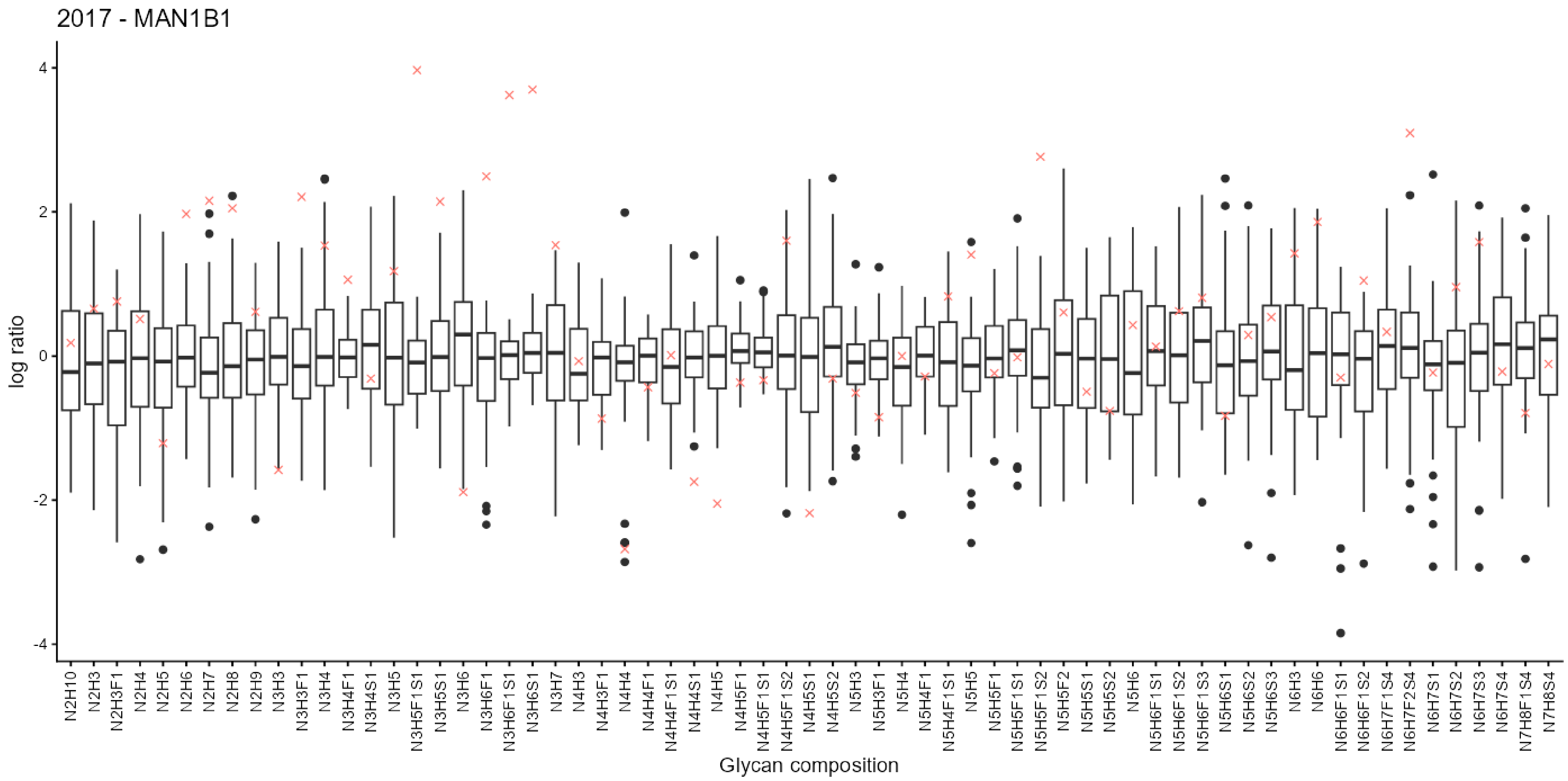

Supplementary Figure S13 Relative quantification of *N*-glycans for patient 2017. Red crosses represent the $\log_{10}$ abundance ratios of N-glycans from the patient relative to the control pool. Black circles indicate individual control values identified as outliers

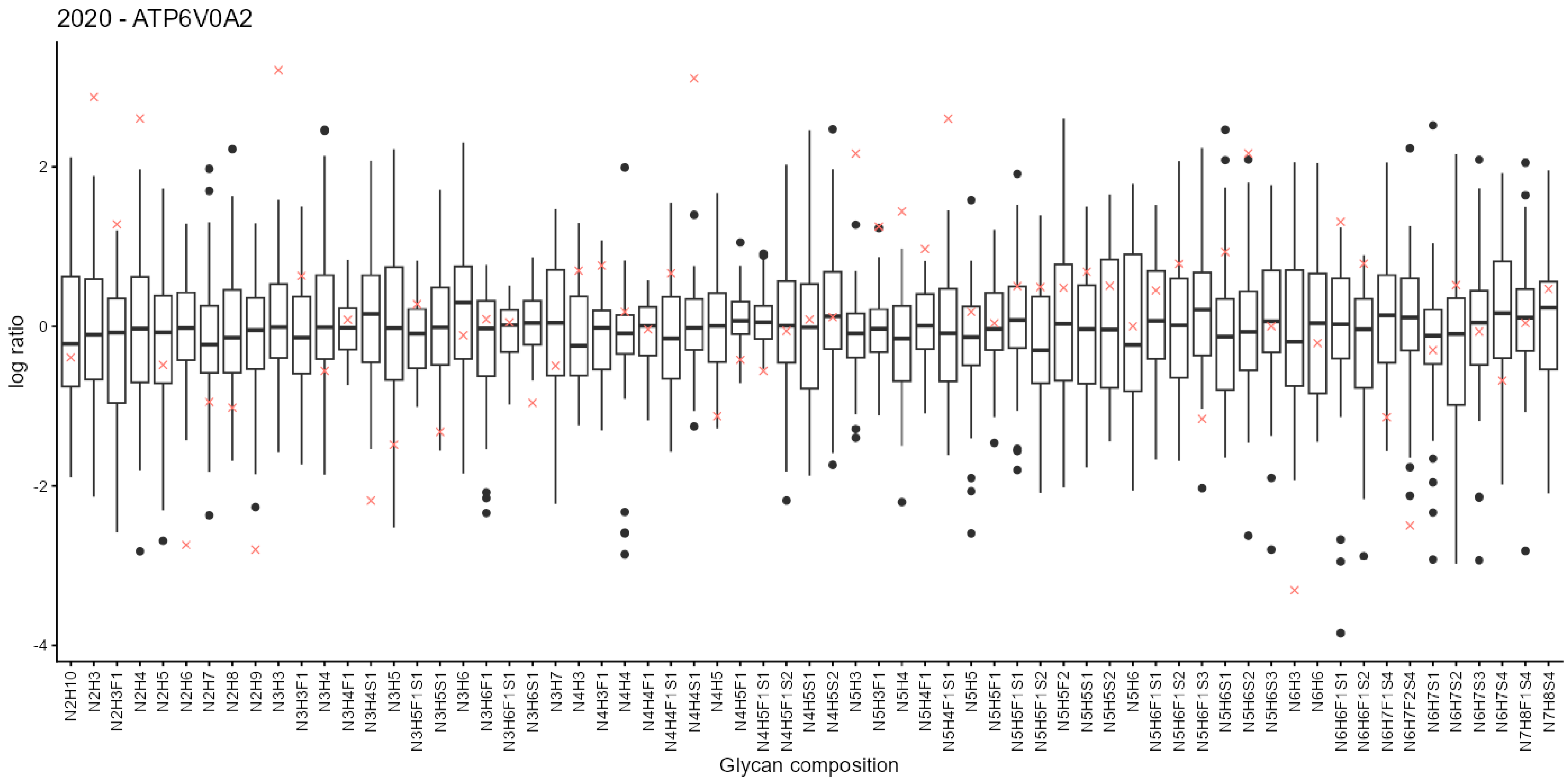

Supplementary Figure S14 Relative quantification of *N*-glycans for patient 2020. Red crosses represent the log_{10} abundance ratios of N-glycans from the patient relative to the control pool. Black circles indicate individual control values identified as outliers

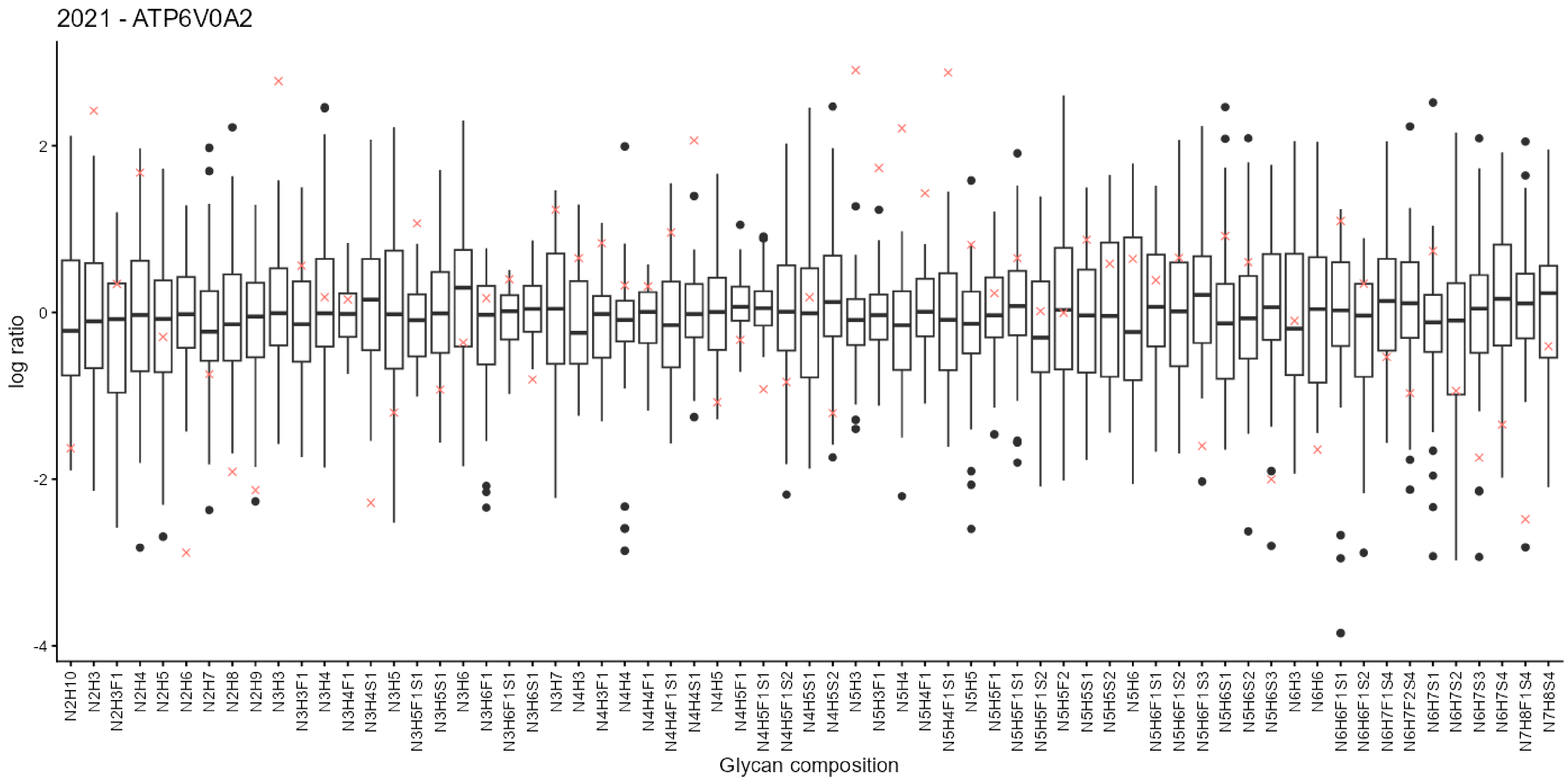

Supplementary Figure S15 Relative quantification of *N*-glycans for patient 2021. Red crosses represent the $\log_{10}$ abundance ratios of N-glycans from the patient relative to the control pool. Black circles indicate individual control values identified as outliers

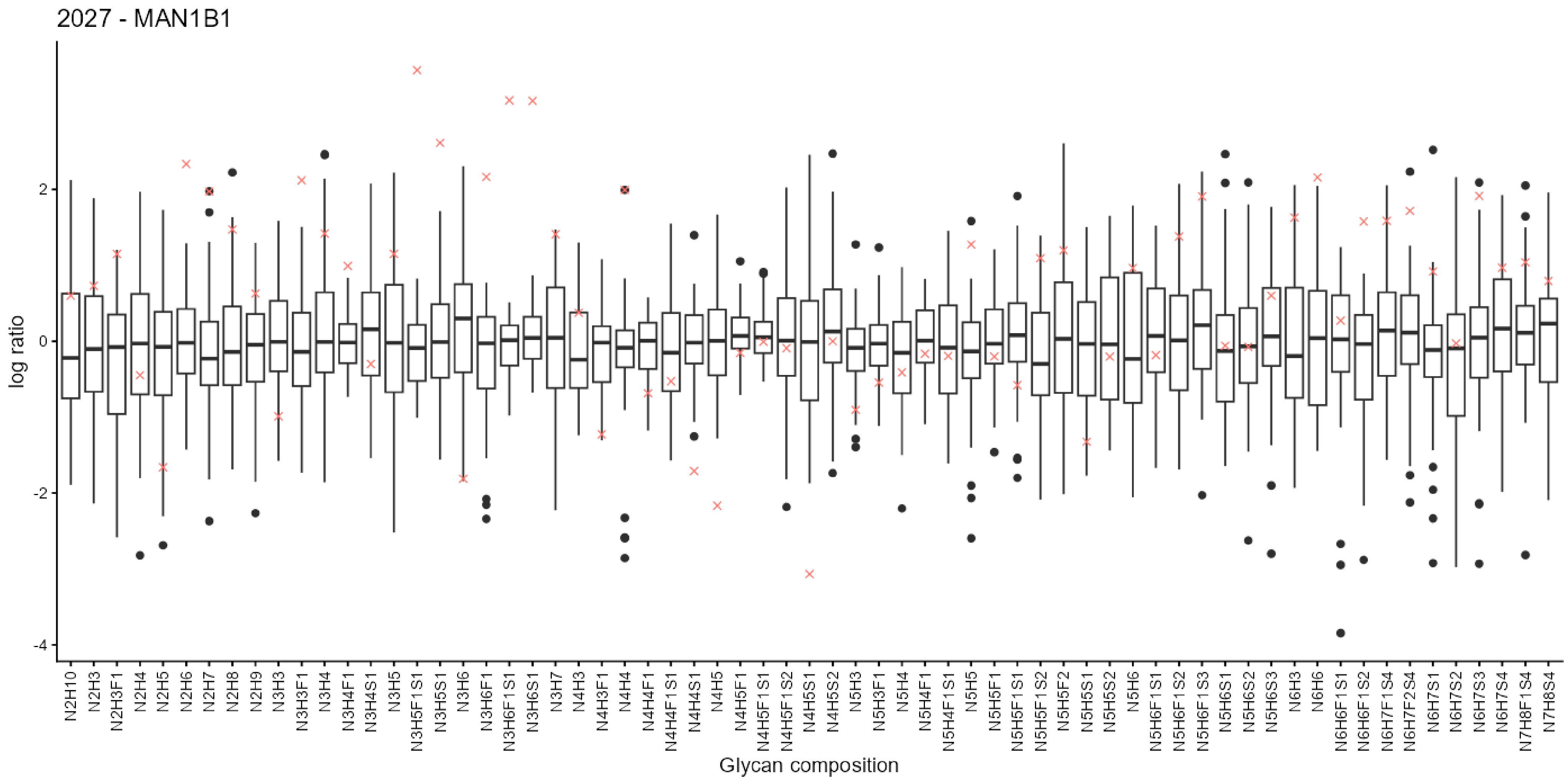

Supplementary Figure S16 Relative quantification of *N*-glycans for patient 2027. Red crosses represent the $\log_{10}$ abundance ratios of N-glycans from the patient relative to the control pool. Black circles indicate individual control values identified as outliers

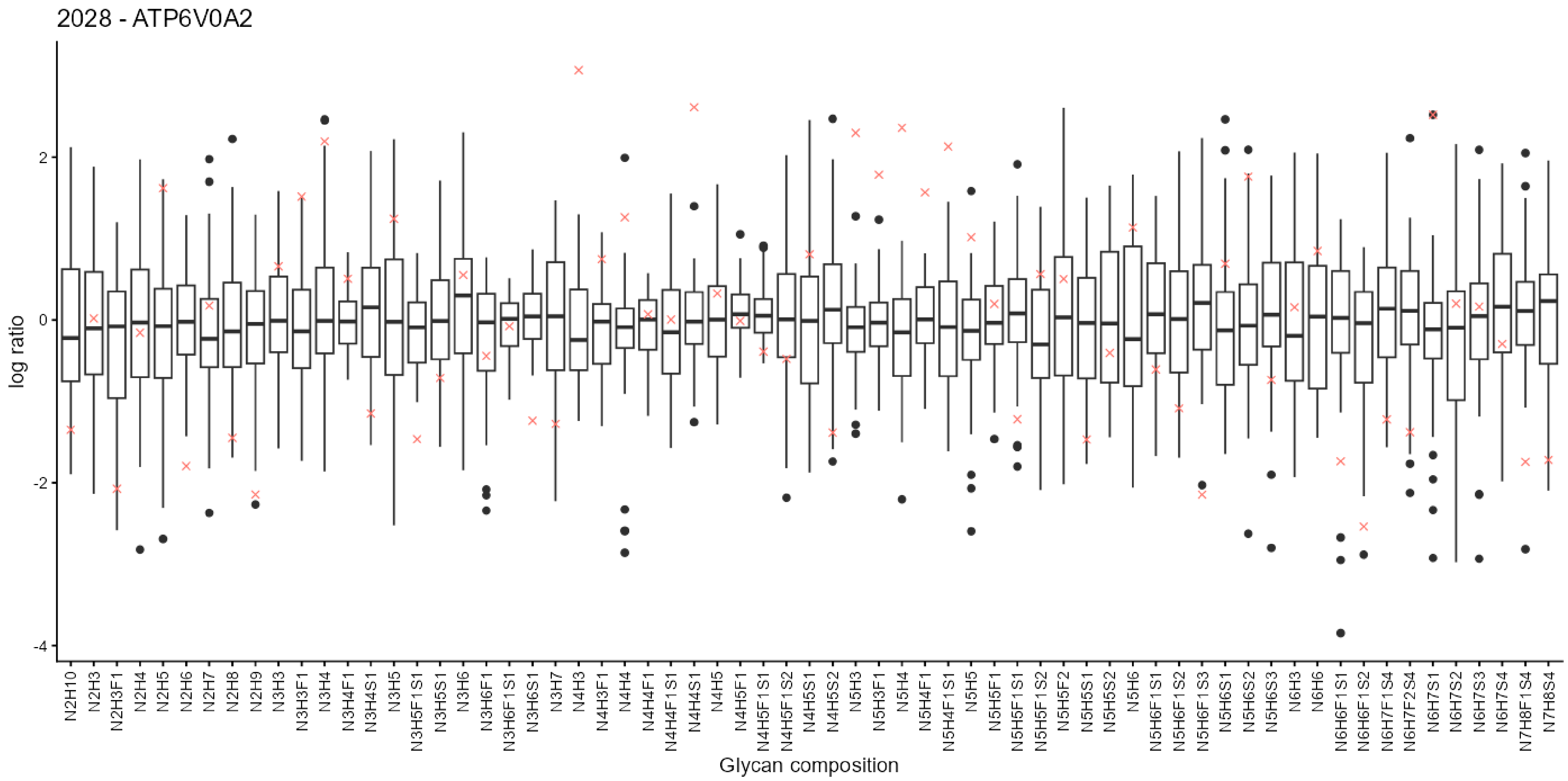

Supplementary Figure S17 Relative quantification of *N*-glycans for patient 2028. Red crosses represent the $\log_{10}$ abundance ratios of N-glycans from the patient relative to the control pool. Black circles indicate individual control values identified as outliers

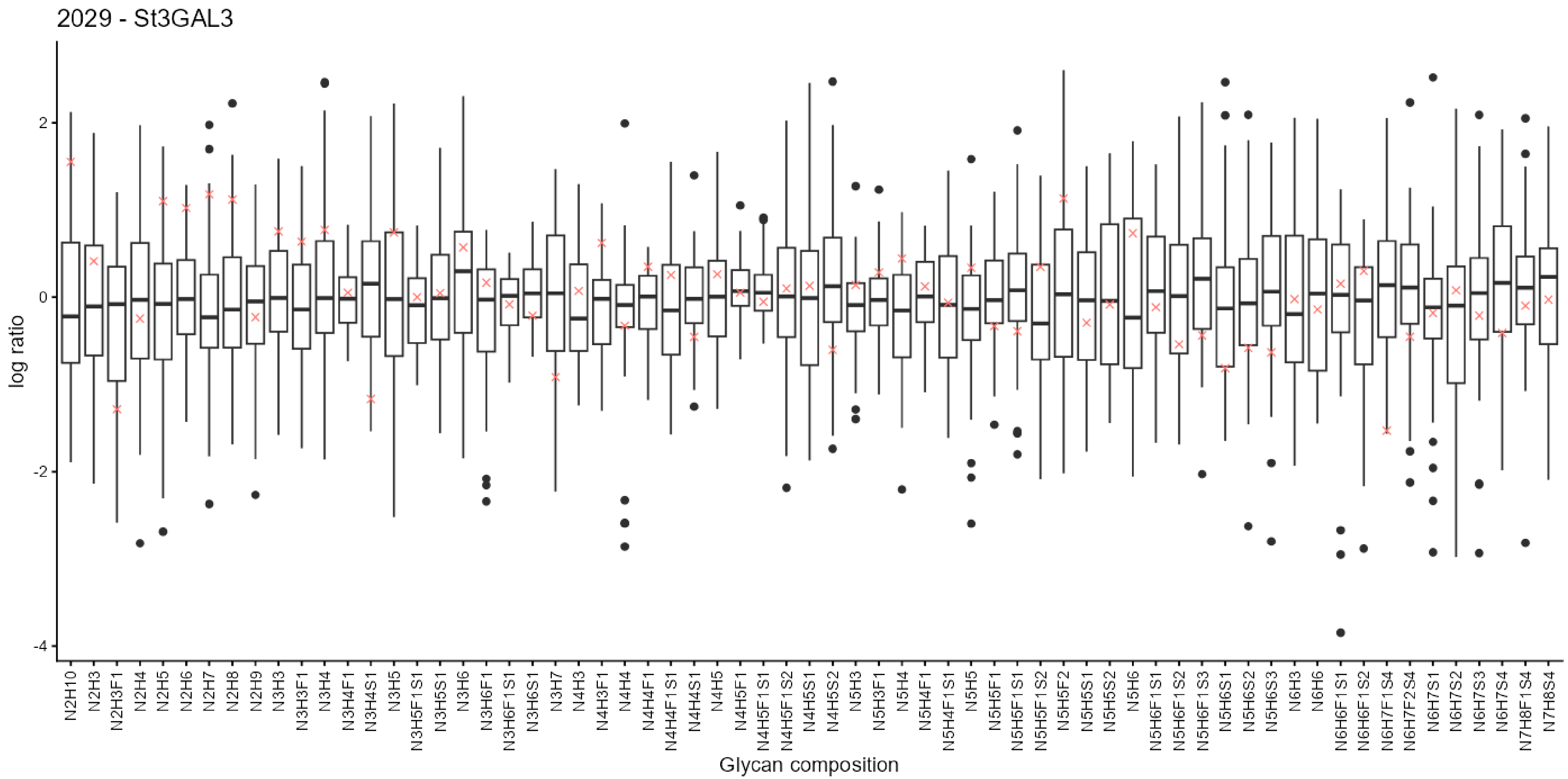

Supplementary Figure S18 Relative quantification of *N*-glycans for patient 2029. Red crosses represent the $\log_{10}$ abundance ratios of N-glycans from the patient relative to the control pool. Black circles indicate individual control values identified as outliers

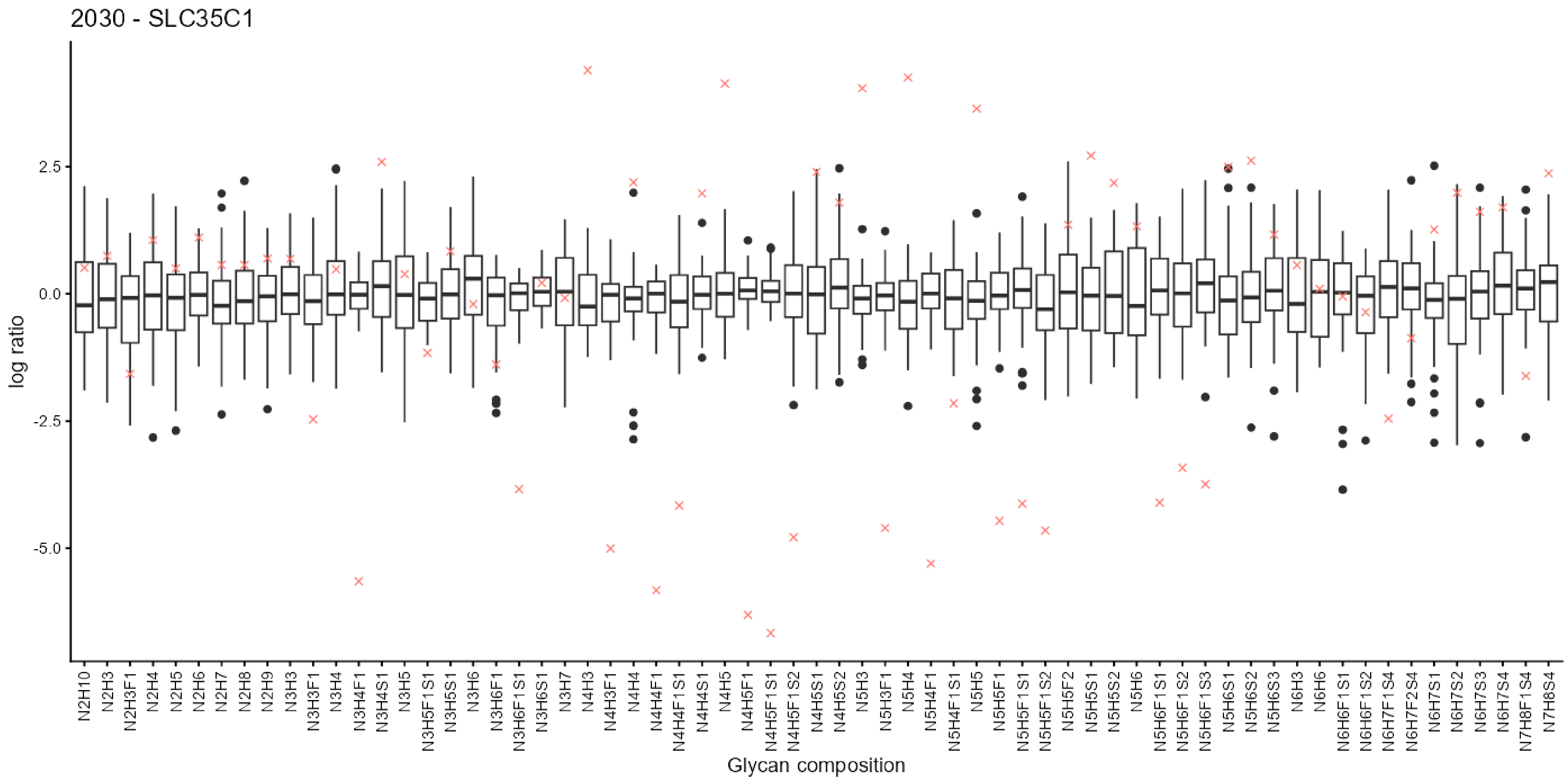

Supplementary Figure S19 Relative quantification of *N*-glycans for patient 2030. Red crosses represent the $\log_{10}$ abundance ratios of N-glycans from the patient relative to the control pool. Black circles indicate individual control values identified as outliers
